# Trigeminal neuralgia and genetics: A systematic review

**DOI:** 10.1177/17448069211016139

**Published:** 2021-05-17

**Authors:** Mari Aaroe Mannerak, Aslan Lashkarivand, Per Kristian Eide

**Affiliations:** 1 Faculty of Medicine, University of Oslo, Oslo, Norway; 2 Department of Neurosurgery, Oslo University Hospital—Rikshospitalet, Oslo, Norway; 3 Institute of Clinical Medicine, Faculty of Medicine, University of Oslo, Oslo, Norway

**Keywords:** Systematic review, familial trigeminal neuralgia, trigeminal neuralgia, trigeminal neuropathic pain, genetics

## Abstract

Trigeminal neuralgia (TN) is a severe facial pain disease of unknown cause and unclear genetic background. To examine the existing knowledge about genetics in TN, we performed a systematic study asking about the prevalence of familial trigeminal neuralgia, and which genes that have been identified in human TN studies and in animal models of trigeminal pain. MedLine, Embase, Cochrane Library and Web of Science were searched from inception to January 2021. 71 studies were included in the systematic review. Currently, few studies provide information about the prevalence of familial TN; the available evidence indicates that about 1–2% of TN cases have the familial form. The available human studies propose the following genes to be possible contributors to development of TN: CACNA1A, CACNA1H, CACNA1F, KCNK1, TRAK1, SCN9A, SCN8A, SCN3A, SCN10A, SCN5A, NTRK1, GABRG1, MPZ gene, MAOA gene and SLC6A4. Their role in familial TN still needs to be addressed. The experimental animal studies suggest an emerging role of genetics in trigeminal pain, though the animal models may be more relevant for trigeminal neuropathic pain than TN per se. In summary, this systematic review suggests a more important role of genetic factors in TN pathogenesis than previously assumed.

## Introduction

Trigeminal neuralgia (TN) is a devastating facial pain disease, typically characterized by severe paroxysmal pain attacks within the innervation area of the fifth cranial nerve (CNV).^1^ In some TN cases no cause may be identified (denoted idiopathic TN), while in others TN may be caused by vascular compression of the trigeminal nerve root (denoted classical TN), or result from lesions affecting the trigeminal nerve, such as tumors, vascular malformations or multiple sclerosis (denoted secondary TN).^2^ It is also common to differentiate between the two phenotypes TN type 1 (TN1) and TN type 2 (TN2), where TN1 is characterized by short-lasting attacks (seconds to few minutes) of sharp, shooting, electrical shock-like pain, and TN2 characterized by constant pain of aching, throbbing, or burning character.^3^ The underlying pathophysiology is not fully understood, but involve both peripheral and central mechanisms.^4^ Focal demyelination of the trigeminal nerve at the root entry zone nearby pons may render the primary afferent neurons hyper-excitable to mechanical compression from e.g. blood vessels in the subarachnoid space.^4^ In particular, voltage gated sodium and potassium channels seem crucial for ectopic activity in trigeminal primary afferents.^5^ Possible central mechanisms are hyper-excitability of 2nd order neurons within the trigeminal brainstem sensory nuclei or within 3rd order neurons in thalamus that project to the cortical grey matter. Neuroimaging studies have provided evidence of changes in brain structure such as grey matter volume and connectivity of TN patients.^5^

The role of genetic factors in the development of TN is unclear, but it was recently suggested to be more important than previously assumed.^6^ To examine the current knowledge about the role of genetic factors and identified genes in TN, we performed a systematic review asking three main questions: 1) What is the prevalence of familial TN among individuals with diagnosed TN? Even though most cases of TN are sporadic, it is known that some are familial.^7^ The occurrence of familial forms of TN is an indicator of genetic involvement in the condition. 2) Which genes have been identified in human studies on TN, both including sporadic and familial forms? 3) Which genes have been identified in animal studies on experimental trigeminal pain? We included animal studies to get a more complete picture of the topic in question, even though the experimental data may be more relevant for trigeminal neuropathic pain than TN per se. Experimental animal studies may, however, provide important information about possible pathophysiological mechanisms behind TN in humans.

## Methods

### Data sources

The review protocol was registered in PROSPERO (CRD42020158967), available from https://www.crd.york.ac.uk/prospero/display_record.php?ID=CRD42020158967.

Prior to the literature searches, we formulated the following research question: “What is the prevalence of familial trigeminal neuralgia and which genes are involved in the development of trigeminal neuralgia?”

To answer this main question, we included the following three categories of studies: 1) Human studies including patients with familial trigeminal neuralgia not restricted by age, gender, ethnicity or comorbidity. 2) Human studies about genetics in individuals diagnosed with TN with no restrictions regarding gender, age, ethnicity or comorbidity. Other trigeminal pain conditions than TN were not included. 3) Animal studies on experimental trigeminal pain, where the trigeminal nerve was manipulated to provoke trigeminal pain. Any study design was to be included in this systematic review. There was no date restriction.

Prior to the literature search, we were aware that the experimental studies in animals address trigeminal pain in general, and not TN specifically, as the latter is difficult to investigate in animal studies. The experimental studies involve manipulation of the trigeminal nerve to provoke trigeminal pain, which may be more relevant for neuropathic trigeminal pain than TN per se. On the other hand, experimental studies may provide crucial information about trigeminal pain mechanisms that may be highly relevant for TN in humans.

Exclusion criteria were as follows: Studies in any other language than English, as well as gray literature, unpublished studies, abstracts and conference presentations.

The decisions on eligibility criteria for inclusion and exclusion were revised and determined by consensus among two of the reviewers (MAM and PKE) based on experience and our preliminary search on this topic.

Search strategy for PubMed, Embase, Cochrane Library and Science Citation Index (Web of Science) was established by an experienced librarian at the Medical Library, University of Oslo. The search was updated on January 11th, 2021. References in review articles were also checked carefully for relevant studies. A full description of our search strategy is attached in Supplementary Material.

### Study selection

A digital library with the retrieved references from the databases was created. Duplicates were removed. Titles and abstracts were then carefully screened independently by two reviewers (MAM and PKE) for selection and inclusion, and human studies not involving data on familial TN or genetic investigation of the disease were excluded. Details on inclusion and exclusion criteria can be found in the review protocol on PROSPERO.

The quality of case reports was assessed according to the CARE guidelines.^8^ The JBI critical appraisal tool was used by the authors.^9^ Due to rarity of the condition and limited reported studies, a predetermined strategy of low threshold for selection and inclusion of studies regarding familial trigeminal neuralgia - or trigeminal neuralgia and genetics, was agreed on by the authors. Nevertheless, a critical assessment of the description of demographic, familial relation and identified genes, patient history and timeline, and a detailed presentation of clinical condition was done by the reviewers. Articles meeting the criteria for inclusion were discussed in detail by the two reviewers until a consensus was achieved. In case of disagreement, the third reviewer (AL) was involved to resolve the dispute. The level of evidence was assessed using the OCEBM (Oxford Centre for Evidence-Based Medicine).^10^ Most studies included in the present systematic review are case reports, cross-sectional studies and experimental animal studies, and were thus assessed as Level 5 studies. A detailed description of the level of evidence is attached as Supplementary material.

### Data extraction

After the assessment of the full texts according to our inclusion criteria, the same two reviewers extracted data from the full texts of the included studies, organized in three different categories, namely (1) Familial TN, (2) Human studies on genetics in TN, and (3) Animal studies on experimental trigeminal pain.

#### Familial TN

For each study in the familial TN group, the following data were extracted: Publication year, number of families with familial TN, number of patients, gender (including female:male ratio), age of TN onset, comorbidity, suggested inheritance pattern, characteristics of TN, estimated prevalence of familial TN and suggested mechanism of the disease (Table 1).

**Table 1.** Studies on occurrence of familial trigeminal neuralgia.

Author, year	Number of families	Gender (F:M ratio*)	Age of TN onset**	Comorbidity	Suggested inheritance pattern	Characteristics of TN***	Estimated prevalence of fam TN	Mechanisms
Mereaux et al.,^24^ 2019	1	4 F (4:0)	Min age 30, max age 51	CMT	AD	1) L V1+V2+V3 and R V3 2) R + L TN	N/A	MPZ mutation
Fernandez Rodriguez et al.,^13^ 2019	5	7 F, 4 M (1,75:1)	62.9 +/- 13.93 y	Arterial hypertension in 1 family	AD with anticipation	V2+V3 (36,3%)R/L ratio 2,67:1	2%	Inherited anatomical conformation of the cranium or familial arterial hypertension resulting in tortuous vessels compressing the nerve
Cervera-Martinez et al.,^27^ 2018	2	2 F, 3 M(2:3)	50 y	Hypertension, atherosclerosis, smoking, umbilical hernia, ASA allergy, smoking	AR, AD, anticipation	1) L V2+V3 2) V3 (side N/A) 3) L V2+V34) R + L (branch N/A)	N/A	SCA in contact with CNV, arachnoid adhesions at the cerebellopontine angle.
Denu et al.,^21^ 2017	1	3 M (0:3)	31 y in 1 patient (two N/A)	Migraine with aura	AD	1) R V1+V2+V3	N/A	Idiopathic
Zhang et al.,^32^ 2016	1	4 F (4:0)	49 y	Glossopharyngeal neuralgia	X-linked or AD	1) R V1+V2+V32) R + L V1+V2+V33) R V2	N/A	Vascular disorders, small posterior cranial fossas, anatomical variations of the posterior circulation, hypersensitivity of cranial nerves and other abnormalities.
Ebner et al.,^15^ 2010	1	3 F, 1 M (3:1)	42 y	Psychological trauma	AD	1) R + L TN (branch N/A)	< 1%	Neurovascular conflict, atherosclerotic vascular changes, abnormalities in myelination and central neuronal hyperactivity.
Savica et al.,^17^ 2007	1	1 F, 3 M (1:3)	63 y	None	AD	1) R V2	1–2%	Genes coding for calcium channels. Familial vascular malformation. Skull base abnormalities.
Smyth et al.,^25^ 2003	1	4 F (4:0)	36,5 y	Hiatal hernia, migraine headaches, hand numbness and tingling	AD	1) V2 (side N/A) 2) L V2+V33) R V24) R V2	N/A	Vascular compression
Gupta et al.,^30^ 2002	1	1 F 2 M (1:2)	47 y	N/A	N/A	1)V1+V22) L V1+V23) R V1+V2	N/A	Vascular compression
Fleetwood et al.,^7^ 2001	1	3 F, 1 M (3:1)	54,8 y	Supraventricular tachycardia, endometriosis	N/A	1) R V2+V32) L V2+V33) L V2+V3, later R V2+V34) L V2+V3	N/A	N/A
Duff et al.,^26^ 1999	1	5 F, 3 M (5:3)	51,4 y	One patient with left HFS, left meningioma and low-grade glioma.	AD, but does not exclude X-linked or mitochondrial heritance	1) R TN (branch N/A) 2) L V2-V33) R V2-V34) R V3, L TN (branch N/A) 5) R + L V26) R V27) R V28) L V2	N/A	Central neuronal hyperexcitability
Coffey et al.,^19^ 1991	1	3 F, 1 M (3:1)	39 y in 1 patient	CMT	AD	1) L V22) L V3, R V2		
Kirkpatrick DB.,^29^ 1989	1	4 F (4:0)	39 y	None	Dominant	1) L V32) L V2	N/A	SCA compressing the TREZSuggesting arteriosclerosis of the vascular network of the posterior fossa with ectasia and pressure on the TREZ in one patient. Suggesting a multifactorial etiology.
Braga et al.,^31^ 1986	1	2 F, 2 M (1:1)	25,8 y	None	N/A	1) V3 (side N/A) 2) R + L V2, but never at the same time3) R + L V2 4) V2 (side N/A)	N/A	Transverse pontine vein and a branch of the petrosal compressing the nerve in one patient
DiCorato, M. P. and Pierce, B. A,^22^ 1985	1	4 F (4:0)	53,3 y	N/A	AD with anticipation	1) L TN (branch N/A) 2) L V1+V2 3) L V1+V24) R TN	N/A	Aneurysm of the internal carotid artery in one patient
Testa^74^ et al., 1981	1	1 F, 2 M (1:2)	40,5 y	CMT	N/A (CMT AD)	1) R V22) R V2+V3, L V1+V2	N/A	Idiopathic
Herzberg, L.,^23^ 1980	1	3 F, 1 M (3:1)	52 y	N/A	AD	1) L V12) L V23) R V14) R V2	N/A	Atrophy of the cerebellar hemisphere with less severe atrophy of the cerebellar vermis in one patient
Knuckey, N. W. and Gubbay, S. S.^75^ 1979	1	2 M (0:2)	1st patient middle 30’s, 2nd patient 50	Left glossopharyngeal neuralgia in 1 patient		1) L V1+V2+V3.	N/A	N/A
Cruse et al.,^28^ 1977	1	4 F, 2 M (2:1)	40,6 y	CMT, deafness	AD with variable penetrance	1) L V22) N/A 3) Unilateral TN (branch N/A) 4) Unilateral TN (branch N/A) 5) L TN (branch N/A) 6) L TN (side N/A)	N/A	Familial hereditary neuropathic disease. Elevated CSF protein level
Daly, R. F. and Sajor, E. E.,^20^ 1973	1	2 F, 2 M (1:1)	36 y	None, 1 patient with slight flattening of nasolabial fold	AD	1) R + L V2+V3 2) R V2+V3, L V23) R TN (branch N/A) 4) L V2+V3	N/A	N/A
Auld et al.,^18^ 1965	1	2 F, 4 M (1:2)	59,8 y	Hemicrania and scotamata. History of seizures	AD	1) R + L V22) R V23) R TN (branch N/A) 4) R + L TN (branch N/A) 6) R V3	N/A	Variation in the anatomy of the middle fossa or some other hereditary variant
Harris, W.,^12^ 1940	N/A	N/A		Disseminated sclerosis	N/A	Bilateral (20%) of those with familial TN	2.1% (30/1433 patients)	N/A
Allan, W.,^16^ 1938	1	1 F, 2 M (1:2)	22 y (one patient), 2 N/A	N/A	AD	1) R TN (branch N/A) 2) R + L TN (branch N/A)	1%	N/A

Abbreviations: N/A, not available; F:M ratio, female/male ratio; F, female; M, male; y, years; TN, trigeminal neuralgia; CNV, fifth cranial nerve; L, left; R, right; V1, ophthalmic branch, V2, maxillary branch; V3, mandibular branch; AD, autosomal dominant; AR, autosomal recessive; SCA, superior cerebellar artery; CMT, Charcot-Marie-Tooth; TREZ, trigeminal root entry zone;.

*F:M ratio = female/male ratio.

** Mean age at TN onset.

***Patient cases are marked with numbers.

#### Human studies on genetics in TN

For the human studies on genetic background of TN we extracted: Publication year, gene, proteins encoded by the genes involved, suggested mechanism, gender, age of TN onset, characteristics of TN, estimated prevalence of familial TN, and comorbidity (Table 2).

**Table 2.** Human studies on genetic background of trigeminal neuralgia.

Author, year	Gene	Proteins encoded by the genes involved	Mechanisms	Gender	Age at TN onset	Characteristics of TN	Estimated prevalence of familial TN	Comorbidity
Gambeta et al.,^36^ 2021	CACNA1A	Calcium channel Ca_V_2.1	Proline 2455 Histidine mutation mediates a depolarizing shift in the voltage-dependence of activation and inactivation. Reduces calcium-dependent inactivation of the channel.	N/A	N/A	N/A	N/A	N/A
Li et al.,^43^ 2020	1) NREP, NACC2, NET1, NCALD, NRG2c.2) NOVA1, NRAS, NF2, NRP2. 3) NGEF, NRG3, NRF2, NFASC, NAV3. 4) NTS, NKAIN2, NDNF, NEUROD4	1) miR-132-3p2) miR-146b-5p3) miR-155-5p4) miR-384	The genes listed in this table are predicted to be targeted by the candidate miRNAs. Reduced proliferation and migration of Schwann cells, inhibition of nerve regeneration and apoptosis. The specific mechanism of differential miRNA expression is still unclear.	F (67,9%)M (32,1%)	N/A	V1+V2 (17,8%)V2 (28,6%)V3 (42,9%)V2+V3 (10,7%)	N/A	N/A
Dong et al.^38^ 2020	GABRG1, SCN8A, SCN5A, CACNA1H, CACNA1F, KCNK1, TRAK1	GABA_A_R Cl^-^ channel, Na_V_1.6, Na_V_1.5, Ca_V_3.2, Ca_V_1.4, K^+^ channel TWIK1, kinesin adaptor protein	Increased sensitivity to the TG or axons to neurovascular compression by an offending blood vessel	F (83,1%)M (16,9%)	N/A	14,5% bilateral TN	N/A	N/A
Di Stefano et al.,^35^ 2020	SCN genes, KCN genes, CACNA genes, CLCN genes, TRP genes CLIC5, GJB5	Na^+^ channels, K^+^ channels, Ca^2+^ channels, Cl^-^ channels TRP channels, and gap junction/connexin channel	Increased neuronal excitability	F (58,33%)M (41,66%)	52 y	V2 (8,3%)V2+V3 (33,3%)V1+V2+V3 (25%), V1-V2 (25%), V3 (8,3%)L TN (0,33%)R TN (0,66%)	11% of the patients reported familial occurrence of TN	Painful neuropathy in one patient
Costa et al.,^33^2019	SCN9A, NTRK1	Na_V_1.7 and Nerve Growth Factor receptor TrkA	rs6746030 polymorphism in SCN9A, rs633 polymorphism in NTRK1. No association was observed between the polymorphisms and TN.	F (56,2%), M (43,8%)	63,7 +/- 12,1 y (age at examination, not age at TN debut)	R TN (66,7%)L TN (27,1%)R + L TN (6,3%)V1 (5,2%)V2 (14, 6%)V3 (10,4%)V1+V2 (4,2%)V1+V3 (6,3%)V2+V3 (45,8%) V1+V2+V3 (14,6%)	N/A	Hypertension (54,2%), diabetes (12,5%), hypercholesterolemia (10,4%), smoking (16,7%)
Caress et al.,^39^ 2019	MPZ Gene	MPZ (Myelin protein zero)	G163T mutation of MPZ gene – protein misfolding resulting in retention of mutant protein	F (100%)	50,8 y	Unilateral (80%) Bilateral (20%)	N/A	CMT 1B, hemifacial spasm
Tanaka et al.,^34^ 2016	SCN8A	Na_V_1.6	Na_V_1.6 mutation reduces the threshold for action potentials in TRG neurons	1 F	63 y	V2	N/A	N/A
Di Lorenzo et al.,^40^ 2014	MAOA gene (X-linked)	MAOA (Monoamine oxidase type A)	MAOA-uVNTR polymorphism (HAM or LAM). Suggesting MAOA as a modulator of neural plasticity related to cortical pain processing	F (50,7%), M (49,3%)	Approximately 30 y	N/A	N/A	N/A
Cui et al.,^41^ 2014	SLC6A4	5-HTT (serotonin transporter)	5-HTTLPR with rs25531 polymorphism is associated with the susceptibility to TN, pain severity of TN and treatment response to CBZ	F (59,0%) M (41%)	55,06 y	V1 (4,1%) V2 (52,0%)V3 (43,9%),	N/A	Current smoking (32,8%), ever smoking (38,5%)
Siqueira et al.,^14^ 2009	N/A	Na_V_1.3, Na_V_1.7, Na_V_1.8	Downregulation of Na_V_1.7, upregulation of Na_V_1.3, no difference in Na_V_1.8	F (60%)M (40%)	46.8+/- 11,4 y	V2 (5 patients) V3 (5 patients)	3%	
Jin et al.,^42^ 2001	N/A	BMP 2, 3, 4 and 5 (Bone morphogenetic proteins)	Finding of BMP 2, 3, 4 and 5 in Schwann cells, and BMP 2 in nerve fibers of the trigeminal nerve in 2 patients with TN	N/A	N/A	N/A	N/A	N/A

Abbreviations: N/A, not available; F, female; M, male; y, years; R, right; L, left; HFS, hemifacial spasm; CMT, Charcot-Marie Tooth; UPR, unfolded protein response.

*Mean age at TN onset.

#### Animal studies on experimental trigeminal pain

The following data were extracted from the experimental studies on genetic background of trigeminal pain in animal models: Publication year, study model, gene, proteins encoded by the genes involved, and suggested mechanism (Table 3).

**Table 3.** Experimental studies genetic background of trigeminal pain in animal models.

Author, year	Study model of trigeminal neuropathic pain	Gene	Protein encoded by the genes involved	Mechanisms
Montera et al.,^56^ 2021	FRICT-ION in mice	Cacna1i	Calcium channel Ca_V_3.3	Cacna1i upregulation as well as protein upregulation of Ca_V_3.3 in TG after FRICT-ION.
Zhao et al.,^76^ 2020	pIONL in mice	Tlr8	TLR8 (Toll-like receptor 8)	Increased TLR8 after pIONL. Deletion of Tlr8 attenuated mechanical allodynia.
Xu et al.,^77^ 2020	CCI-ION in mice	Gm14461 gene, TNF gene, IL genes, CGRP gene, P2rx3, P2rx7	Long non-coding RNA Gm14461, TNF-α, IL-1β, IL-6, CGRP, P2X3/7 receptor	Increased Gm14461, and overexpression of Gm14461 upregulated mRNA levels of TNF-α, IL-1β, IL-6, CGRP and P2X3/7 receptor. Gm14461 promoted pain transmission, and the underlying mechanisms might involve the regulation of pro-inflammatory cytokines, CGRP and P2X3/7 receptor.
Liu et al.,^61^ 2020	CCI-ION in rats	Scn3a, IL-6 gene	Sodium channel Na_V_1.3, IL-6	Increase of Na_V_1.3 and IL-6 in TG. Emergence of Na_V_1.3 from the compressed CNV might be an important structural basis for the development of the ectopic excitability on the axon and IL-6 may play a role of necessary precondition.
Li, et al.,^78^ 2020	pT-ION in mice	Gjd2 gene	Cx36 (Connexin 36)	Upregulation of Cx36, GluK2, TRPA1 and p-ERK in TG after pT-ION. Cx36 contributes to development of orofacial pain hypersensitivity through GluK2, TRPA1 and p-ERK signaling.
Korczeniewska et al.,^79^ 2020	CCI-ION in rats	Long list of genes. Cnr2, Grm5, Htr1a, Il10, Oprd1, Pdyn, Prok2, Tacr1, Adora1, Cd200, Comt, Maob, Mapk3, P2rx4, Ptger1, Tnf, Slc6a2		Cnr2, Grm5, Htr1a, Il10, Oprd1, Pdyn, Prok2 and Tacr1 were upregulated in TG but downregulated in DRG 4 days post-injury. Adora1, Cd200, Comt, Maob, Mapk3, P2rx4, Ptger1, Tnf and Slc6a2 were upregulated in TG but downregulated in DRG 21 days post-injury. The findings suggest that spinal and trigeminal neuropathies due to trauma are differentially regulated.
Jiang et al.,^80^ 2020	pT-ION in mice	Gpr151 gene, neuroinflammation-related genes	Gpr151 GPCR (G-protein coupled receptor), chemokines CCL5, CCL7, CXCL9, CXCL10	Gpr151 was the most significantly upregulated GPCR after pT-ION. Global mutation or knockdown of Gpr151 in TG attenuated mechanical allodynia. There was also an upregulation of neuroinflammation-related genes, including chemokines CCL6, CCL7, CXCL9 and CXCL10 after pT-ION.
Dong et al.^38^ 2020	Gabrg1 knock-in mice	Gabrg1	GABAA receptor Cl^_^ channel	Mice engineered with mutation in Gabrg1 exhibited trigeminal mechanical allodynia and pain-like behavior.
Cui et al.,^57^ 2020	pT-ION in mice	Tacr3	Neurokinin 3 receptor (NK3R, tachykinin family)	Downregulation of Tacr3 in the lateral habenula (LHb) plays a protective role in treating trigeminal nerve injury-induced allodynia by suppressing the hyperexcitability in LhB neurons.
Cui et a.l,^81^ 2020	pT-ION in mice	Cacna2d1, Prkca gene, Trpa1 gene, Gjb2, Gjd2, Gja1	Calcium channel subunit Ca_V_α2δ1, PKC (protein kinase C), TRPA1 (transient receptor potential ankyrin 1), Cx(connexin)26, Cx36, Cx43	Ca_V_α2δ1 overexpression in TG neurons induced upregulation of PKC and TRPA1, concluding that Ca_V_α2δ1 contributes to development of hyperalgesia through PKC-TRPA1/Gap junction/connexin signaling.
Aczel et al.,^58^ 2020	CFA-injection in mice	Tac4	Hemokinin-1 (HK-1, tachykinin family)	Upregulation of Tac4 in TG and in SGC in response to inflammation. It is concluded that HK-1 may participate in neuron-glia interactions under physiological and inflammatory conditions and mediate pain in the trigeminal system.
Wang et al.,^82^ 2019	CCI-ION in rats	PTCH gene	Patched1	Patched1 decrease and miR-195 increase. The results suggest that miR-195 is involved in development of TN by targeting Patched1 in the Shh signaling pathway, thus regulating extracellular glutamate.
Lin et al.,^68^ 2019	CFA-injection in rats	P2ry14 gene	P2Y_14_ receptor (purinergic receptor)	Upregulation of P2Y_14_ receptor, glial fibrillary acidic protein (GFAP), interleukin-1β (IL-1β), tumor necrosis factor-α (TNF-α), C-C chemokine CCL2, phosphorylated extracellular signal-regulated kinase 1/2 (p-ERK1/2) and phosphorylated p38 (p-p38) proteins in the TG. P2Y_14_ receptor antagonist attenuated pain, and also decreased the upregulation of GFAP, IL-1β, TNF-α, CCL2, p-ERK1/2 and p-p38 proteins. P2Y_14_ receptor in TG may contribute to orofacial inflammatory pain via regulating SGCs activation, releasing cytokines and phosphorylating ERK1/2 and p38.
Li et al.,^53^ 2019	CCI-ION in rats	Kcnk18 gene	TRESK (TWIK-related spinal cord K+) channel	mRNA and protein levels of TRESK were downregulated after CCI-ION. Treatment with TRESK reduced mechanical allodynia.
Li et al.,^83^ 2019	Incorrectly positioned dental implants in rats to induce trigeminal neuropathic pain	JAK2 gene, PTEN gene	JAK2, PTEN	Increased expression of JAK2 and PTEN. AKT (downstream of PTEN) was downregulated. JAK2 and PTEN may regulate inflammatory responses of nerves and development of neuropathic pain.
Lee et al.,^84^ 2019	Malpositioned dental implant to induce inferior alveolar nerve injury in rats	VEGFA gene	Vascular endothelial growth factor-A (VEGF-A)	Upregulation of astrocytic VEGF-A in the medullary dorsal horn following nerve injury. The allodynia was inhibited by VEGF-A_164_ antibody.
Guo et al.,^54^ 2019	TRESK knock-out mice	Kcnk18 gene	TRESK (TWIK-related spinal cord K+) channel	Loss of TRESK in TG neurons increased the excitability of nociceptors, increasing the chances of developing headache.
Liu et al.,^50^ 2018	Dural infusions to induce rat models of trigeminal allodynia	P2x4r gene	P2X purinoceptor 4 (ligand-gated cation channel activated by extracellular ATP)	Upregulation of P2X4R. Blockage of the receptor produced an anti-nociceptive effect.
Korczeniewska et al.,^45^ 2018	CCI-ION in rats, selected gene expression changes were examined with real-time PCR	Long list of genes. Kcnip3, Kcnj6, Kcnq2, Kcnq3, Scn10a, Scn9a, Oprd1, Oprm1, P2rx3, P2ry1, Trpa1, Trpv1, Cnr1, Calca, Cckbr, Chrna4, Tac1, Tacr1, Ptger3, Ptger4, Ntrk1, Bdnf, Grin1, Htr1a, Htr2a, Cnr2, Ccr2, Cd4, Csf1, Cx3cr1, Itgam, Itgb2, Tlr2, Adrb2 and more	K^+^ channels, Na^+^ channels, opioid receptors, purinergic receptors, cannabinoid receptors, TRP channels, purinoceptors, inflammatory regulation system proteins, elcosanoid metabolism proteins, neurotrophin pain response modulation system proteins, glutamate receptor, serotonin receptor, pain conduction protein, inflammatory pain response modulation proteins, neurotransmitter regulation protein	Downregulated genes: Kcnip3, Kcnj6, Kcnq2, Kcnq3 (potassium channels), Scn10a, Scn11a, Scn9a (sodium channels), Oprd1, Oprm1 (opioid receptors), P2rx3, P2ry1 (purinergic receptors), Trpa1, Trpv1 (ion channels), Cnr1 (cannabinoid receptors), Calca, Cckbr, Chrna4, Tac1, Tacr1 (inflammatory regulation system), Ptger3, Ptger4 (eicosanoid metabolism), Ntrk1, Bdnf (neurotrophin pain response modulation system), Grin1 (glutamate receptor gene), Htr1a, Htr2a (serotonin receptor genes). Upregulated genes: Cnr2 (pain conduction), Ccr2, Cd4, Csf1, Cx3cr1, Itgam, Itgb2, Tlr2 (inflammatory pain response modulation), Adrb2 (neurotransmitter regulation system). Females developed more allodynia but not hyperalgesia compared to males. Cck, Il1a, Pla2glb and Tnf genes regulated in females but not in males. Chrna4 gene downregulated in males but not in females.
Demartini et al.,^49^ 2018	CCI-ION in rats	Trpv1 gene, Trpa1 gene, Calca, PPT-A	TRPV1 (TRP vanilloid type-1) channel and TRPA1 (transient receptor potential ankyrine type-1) channel, CGRP (calcitonin-related polypeptide alpha) and preprotachykinin-A	Increased expression of Trpa1, Trpv1, Calca and PPT-A mRNA in the ipsilateral TG and cervical spinal cord. IL-1β, IL-16 and TNF-α mRNA expression levels were increased in the same areas. ADM_12 treatment reversed these changes, and also reduced the mechanical responses.
Chen et al.,^85^ 2018	CFA-injection in mice	NRP3 gene	NOD-like receptor family pyrin domain containing 3	Increased mRNA expression of NLRP3, IL-1β, IL-18 in TG. Differential expression of 26 miRS. Expression of miR-186 showed the lowest level of all miRs. The expression of NLRP3, IL-1β and IL-18 in TGs were inhibited by miR-186 mimics treatment. miR-186 was able to suppress the neuropathic pain via regulating the NLRP3 inflammasome signaling.
Aczel et al.,^86^ 2018	CFA-injection in rats	List of genes. Kiss1 gene, Gpr39 gene, Lkaaear1 gene, Neurod2 gene	Kisspeptin-1, Kisspeptin-1 receptor, Gpr39 (G-protein coupled receptor 39), Lkaaear, Neurod2	512 differentially expressed genes. Upregulation of Gpr39 and Lkaaear1, downregulation of Kiss1, Kiss1r and Neurod2 in TG.
Benedet et al.,^87^ 2017	Transgene mice (DREAM knock-out mice)	Kcnip3 gene. Pdyn and BDNF genes.	DREAM (downstream regulatory element antagonist modulator) protein = KChIP3 = calsenilin. Prodynorphin and brain-derived neuroptrophic factor.	Reduced expression of DREAM, Pdyn and BDNF in TG, thereby increased responses in transgene mice.
Xu, W et al.,^44^ 2016	CCI-ION in rats	Scn3a, Scn9a, Scn10a, Scn11a	Sodium channels Na_V_1.3, Na_V_1.7, Na_V_1.8, Na_V_1.9	Upregulation of Na_V_1.3. Downregulation of Na_V_1.7, Na_V_1.8, Na_V_1.9.
Trevisan et al.,^48^ 2016	CCI-ION in mice (mice controls, Trpa1 positive and Trpa1 negative)	Trpa1 gene	TRPA1 (transient receptor potential ankyrin 1) channel	Trpa^+^ mice had a lower pain threshold after CCI-ION. Genetic deletion of Trpa1 reduced pain.
Rozas et al.,^66^ 2016	Transgenic mice that overexpress TNF-α	TNF gene	TNF-α	TNF-α regulated Cdk5 activity in TG, inducing sensitization of the TRPV1 channel.
Hanstein et al.,^88^ 2016	Injection of CFA in transgenic animals with Panx1 deletion	Panx1 gene	Pannexin1	Deletion or blockade of Pannexin1 prevents allodynia. Panx1 expression and function are increased during allodynia.
Daiutolo et al.,^89^ 2016	CCI-ION in mice	iNOS gene, CGRP gene	Nitric oxide synthase (NOS), calcitonin gene-related peptide (CGRP)	Focal injury to the sensory cortex increases transcription and protein synthesis of iNOS. Increased level of CGRP in the ascending trigeminal pathway.
Luiz et al.,^90^ 2015	CCI-ION in mice	Bdkrb1 and Bbkrb2 gene	Kinin B1, Kinin B2	Treatment with B1 and B2 antagonist reduced allodynia. Knock-out mice for kinin B1, B2 or both did not develop hyperalgesia. Anti-dynorphin A reduced heat hyperalgesia.
Luiz et al. ^46^ 2015	CCI-ION in mice	Scn11a	Sodium channel Na_V_1.9	Na_V_1.9 knock-out mice did not develop hypersensitivity after CCI-ION. No change in expression of Na_V_1.9 mRNA in TG.
Liu et al.,^55^ 2015	CCI-ION in rats	Bkca gene	BKCa (calcium-activated potassium channel)	Downregulation of BKCa in ipsilateral TG. Increased levels of ERK (extracellular signal-regulated kinase), p38 and JNK (c-Jun N-terminal kinases) in TG, all known to have different functions in sensory processing.
Tzabazis et al.,^91^ 2014	Injection of CFA or formalin in mice. Injection of viral vector encoding for encephalin (SHPE) to induce expression of the transgene	Penk gene	Preproenkephalin	Analgesic effect after SHPE injection. SHPE injection induced expression of the enkephalin transgene in trigeminal neurons.
Chen et al.,^47^ 2014	Formalin induced pain in mice	Trpv4 gene, Trpa1 gene	TRPV4 and TRPA1 ion channels	TRPV4 and TRPA1 important for pain transmission. Knock-out mice for both genes and inhibitors of the receptors attenuated pain.
Poh et al.,^92^ 2012	Facial carrageenan injection in mice		S100a8, S100a9, Lcn2, Il2rg, Fcgr1, C1qb, Ptprc, Ccl12, Cd52	Upregulation of S100a8, S100a9, Lcn2, Il2rg, Fcgr1, C1qb, Ptprc, Ccl12, Cd52 in PFCTX and in blood.
Ma et al., ^93^ 2012	CCI-ION in transgenic rats (Sleeping Beauty transposon mutation for neuregulin 1 transgene)	Neuregulin 1 gene (Nrg1)	Neuregulin 1	No development of trigeminal neuropathic pain in transgenic rats.
Miyamoto et al.^52^ 2011	pT-ION in mice	GluR2, Glur3 gene	Glur2 and Glur3 subunits of the AMPA receptor	No nociceptive behavior in Glur2/Glur3 knock-out mice.
Aita et al.^94^ 2010	pIONL in KOR gene deleted mice	Kor gene	Dynorphin-kappa opioid receptor (KOR)	Enhanced allodynia in KOR gene deleted mice.
Vit et al.,^51^ 2008	CCI-ION in rats	Kcnj10	Potassium channel Kir4.1	Silencing of Kir4.1 led to pain-like behavior. Kir4.1 in TG reduced after CCI-ION.

Abbreviations: N/A, not available; FRICT-ION, foramen rotundum inflammatory constriction of the trigeminal infraorbital nerve; pIONL, partial infraorbital nerve ligation; pT-ION, partial transection of the infraorbital nerve; SGC, satellite glial cells; CCI-ION, chronic constriction injury of the infraorbital nerve; TG, trigeminal ganglion/-a; DRG, dorsal root ganglion/-a; CFA, Complete Freund’s Adjuvant; PFCTX, prefrontal cortex.

### Analyses

The analysis was performed separately for the three different study groups: (1) Studies on familial TN. (2) Human studies on genetics in TN. (3) Experimental studies on genetic background of trigeminal pain in animal models. A meta-analysis was not to be conducted as we included all study designs.

## Results

### Study selection

**Figure 1.**
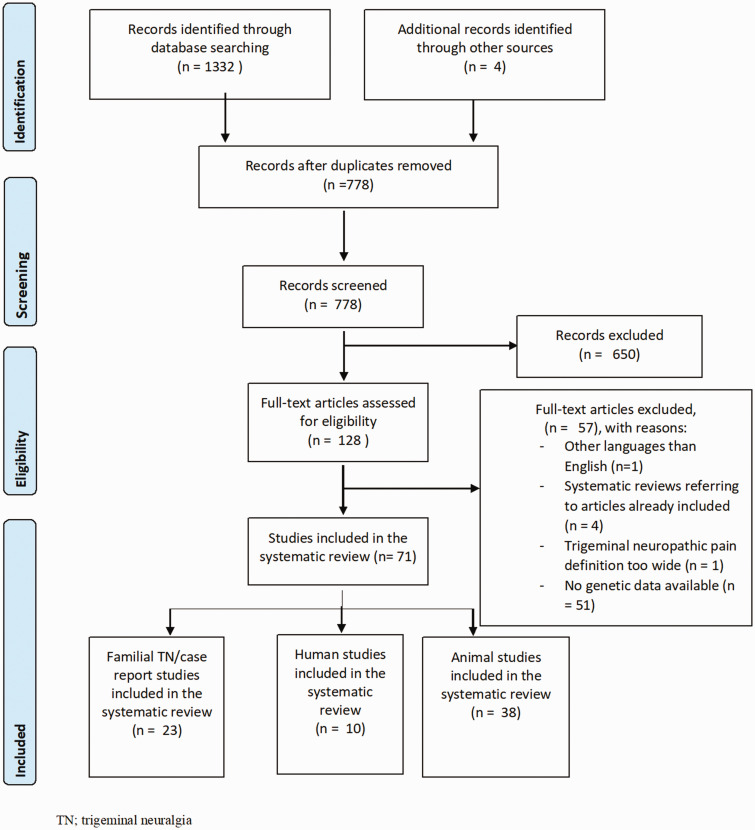
PRISMA flow diagram of included studies.^11^

The Preferred Reporting Items for Systematic Reviews and Meta-Analyses (PRISMA) was used for this review (see Figure 1. and PRISMA checklist included in Supplementary material).^11^ As indicated in Figure 1, 1332 studies were identified from the database search. After removal of duplicates, 774 articles remained. Four other references were included based on findings in review articles, resulting in a total of 778 articles. After excluding non-relevant articles based on titles and abstracts, 128 full-text articles were assessed for eligibility. Following our inclusion and exclusion criteria, 71 of the remaining references were included for this systematic review; 23 case report studies, 10 human studies and 38 experimental animal studies.

### Familial TN

Table 1 provides an overview of included studies regarding familial TN. In the case reports, 27 families with a total of 98 patients were described. Of these patients 60 were women (61.2%) and 38 were men (38.8%). Harris^12^ describes an additional population pool of 30 patients with TN in which heredity of the disease played a part, but no further information regarding number of families, age, sex or inheritance pattern were given. Among individuals with diagnosed TN, the prevalence of familial trigeminal neuralgia is about 1–2%.^12–17^ Mean age of TN onset in the different studies ranged from 22 years ^16^ (lowest mean age) to 63 years ^17^ (highest mean age). Most of the studies suggests an autosomal dominant (AD) inheritance pattern,^15–26^ some even with genetic anticipation phenomenon.^2,13,22,27^ Cruse et al.^28^ suggest AD inheritance with variable penetrance, while Kirkpatrick D. B.^29^ suggests a dominant pattern of genetic transfer without any further specification. Vascular compression is the most frequent presumed mechanism,^25,27,29–31^ while the remaining studies suggest other vascular disorders,^17,22,32^ MPZ mutation,^24^ mutations in calcium channel coding genes,^17^ inherited anatomical abnormalities,^17,18,23,32^ arachnoid adhesions,^27^ central neuronal hyperexcitability^15,26^ and familial hereditary neuropathic disease (Charcot-Marie-Tooth)^28^ as possible explanations of disease development. Fernandez Rodriguez et al.^13^ introduce one family with arterial hypertension and explains that familial hypertension – resulting in tortuous vessels – combined with anatomical conformation at the base of the cranium might lead to compression of the nerve and thereby development of TN.

### Human studies on genetics in TN

Ten human studies investigated the genetic background of the disease in TN patients (Table 2). Several possible genes were identified.

#### Ion channels

Costa et al.^33^ investigated whether there is an association between certain polymorphisms in SCN9A (gene coding for sodium channel Na_V_1.7) and NTRK1 (gene coding for Nerve Growth Factor receptor TrkA). There was no association between the two polymorphisms and TN, but they could not exclude a possible association between other genotypes and TN. Other studies also investigated the possible association between sodium channel genes and development of TN. Tanaka et al.^34^ suggest that a Met136Val mutation in SCN8A (gene coding for sodium channel Na_V_1.6) increases the excitability of the trigeminal ganglion (TG), and thereby reduces the threshold for action potentials in TG neurons. Siqueira et al.^14^ observed downregulation of Na_V_1.7 and upregulation of Na_V_1.3 in patients with TN. The authors suggested that TN, at least in part, may be considered a channelopathy. First, demyelination could induce membrane injury of small myelinated (Aδ) fibers that conduct fast pain, and thus upregulate Nav1.3/downregulate Nav1.7. Second, there could be epigenetic, genetic and/or modulating factors, which affect the function of Nav1.7.

Recent studies have investigated the role of calcium channel gene mutations in TN patients. Two studies propose that CACNA gene mutations (mutations in genes coding for calcium channels) are related to neuronal excitability, thus contributing to TN susceptibility or development.^35,36^ CACNA1A gene mutation alters the gating properties of the channel, suggesting that associated changes in the Cav2.1-dependent synaptic communication in the trigeminal system may contribute to the development of TN.

Mutations in sodium channel genes, transient receptor potential (TRP) genes, potassium channel genes and chloride channel genes in patients with trigeminal neuralgia were identified in another study.^35^ A gain of function mutation in SCN10A was detected in in familial occurrence of TN in the same study. The same mutation has previously been reported in a patient with painful peripheral neuropathy.^37^ Voltage-clamp analysis revealed that this mutation shifts channel activation in a hyperpolarizing direction.^35^ Yet another study identified mutations in several ion channel-coding genes in TN patients, more specifically mutations in genes coding for sodium channels, calcium channels and potassium channels.^38^ CACNA1H mutation is one of the genetic mutations being discussed in this study, where the authors describe that the Cav3.2 α-1H channel subunits are activated during GABA_A_R-mediated depolarization and trigger action potentials in sensory neurons. Moreover, they explain that increased expression of Cav3.2 in damaged DRG neurons contributes to the development of neuropathic pain after nerve injury. The authors have several hypotheses on how the different mutations may contribute to TN pathology. First, they suggest a “genetic-mechanical” model in which a germline mutation increases the sensitivity of the trigeminal ganglia or axons to neurovascular compression by blood vessels. Interestingly, they also suggest that mutations could predispose patients to development of bilateral symptoms. Second, germline mutations are suggested to predispose individuals to later-onset TN from a second mutation in the other allele of the same, or another gene in TG neurons or other neurons in the trigeminal system.

#### MPZ mutation

The MPZ gene is coding for myelin protein zero, and a G163T mutation in the gene was investigated in one study^39^ of patients with Charcot-Marie-Tooth (CMT). Of 27 individuals with the mutation, two had TN and three had both hemifacial spasm (HFS) and TN. One of the patients had bilateral TN. According to the authors, MPZ mutations are associated with CMT. MPZ is probably not directly associated with development of TN, but indirectly because CMT patients are more susceptible to development of TN.

#### MAOA gene

Di Lorenzo et al.^40^ investigated MAOA gene polymorphisms and suggest monoamine oxidase type A (MAOA) as a modulator of neural plasticity related to cortical pain processing, by influencing the brain response in a repeated trigeminal electric pain-related evoked potential paradigm.

#### Serotonin transporter

A polymorphism of SLC6A4, coding for 5-HTT (serotonin transporter), is according to Cui et al.^41^ associated with susceptibility to TN, pain severity of TN and treatment response to carbamazepine. The TN patients had a higher prevalence of a specific serotonin transporter genotype than controls.

#### Bone morphogenetic proteins

Jin et al.^42^ found bone morphogenetic proteins (BMP) 2, 3, 4 and 5 in Schwann cells and BMP 2 in nerve fibers of the fifth cranial nerve (CNV) in two patients with trigeminal neuralgia, suggesting the cytokines may contribute to disease development.

#### miRNAs

Lastly, microRNAs (miRNAs) are suggested to be related to the occurrence and development of TN, and four candidate miRNAs are presented in a study by Li et al.^43^ The authors present several genes predicted to be targeted by the candidate miRNAs, all listed in Table 2.

### Animal studies on experimental trigeminal pain

As presented in Table 3, 38 experimental animal studies were included in this systematic review. The most frequent study model is chronic constriction injury of the infraorbital nerve (CCI-ION) in rats or mice inducing experimental trigeminal pain, followed by behavioral testing as a measure of trigeminal pain. Other manipulations of the CNV as a model of trigeminal pain were also seen. Knock-out or transgenic mice or rats were also used in some of the studies to investigate whether the genetic technique had a connection to either trigeminal pain development or pain relief. The experimental trigeminal pain is probably more associated with trigeminal neuropathic pain than TN, but may be important to consider when examining the genetic background of TN. A wide range of genes have been identified with regard to experimental trigeminal pain.

#### Ion channels

Sodium channel genes were investigated in several studies. In a study by Xu et al.,^44^ following genes were researched: Scn3a (coding for Na_V_1.3), Scn9a (coding for Na_V_1.7), Scn10a (coding for Na_V_1.8) and Scn11a (coding for Na_V_1.9). Following CCI-ION, the researchers observed upregulation of sodium channel Na_V_1.3, and downregulation of Na_V_1.7, Na_V_1.8 and Na_V_1.9. Similar results were presented in another study,^45^ where Scn10a (Na_V_1.8), Scn11a (Na_V_1.9) and Scn9a (Na_V_1.7) were downregulated in rats after CCI-ION. In another study,^46^ Scn11a knock-out mice did not develop hypersensitivity after CCI-ION, although there was no change in Na_V_1.9 mRNA expression level.

Chen et al.^47^ observed that TRPV4 and TRPA1 ion channels are important for pain transmission, and that knock-out mice for both genes resulted in pain attenuation. Trevisan et al.^48^ present similar results where Trpa1^+^ mice had a lower pain threshold, and mice with genetic deletion of Trpa1 experienced pain relief. Increased expression of Trpa1 and Trpv1 was observed in another study^49^ following CCI-ION in rats.

P2x4r gene, coding for P2X purinoceptor 4 – a ligand-gated cation channel – was investigated in a study by Liu et al.^50^ Manipulation of the nerve resulted in upregulation of P2x4r, and blockage of the receptor showed antinociceptive effect.

Kir4.1 potassium channel, coded by Kcnj10, was in one study^51^ reduced after CCI-ION in rats. Silencing of Kir4.1 led to pain-like behavior. The AMPA receptor, which is both a glutamate receptor and cation channel, was investigated in a study by Miyamoto et al.^52^ Knock-out mice of the Glur2 and Glur3 subunits of the AMPA receptor attenuated trigeminal neuropathic pain, as well as Kir4.1 was reduced in TG after CCI-ION. Potassium channel genes Kcnip3, Kcnj6, Kcnq2 and Kcnq3 were downregulated after CCI-ION in rats in a study by Korczeniewska et al.^45^ Kcnk18 gene, coding for the TWIK-related spinal cord K^+^ channel (TRESK), was investigated in two separate studies.^53,54^ Both studies concluded that TRESK attenuates trigeminal neuropathic pain, and that loss of TRESK in TG neurons increases the chances of developing pain. Liu et al.^55^ concluded that the calcium-activated potassium channel BKCa might be a potential therapeutic target for trigeminal neuropathic pain.

As well as investigation of calcium channel coding genes in human studies, one recent experimental animal study^56^ investigated the role of calcium channels in experimental trigeminal neuropathic pain models. Cacna1i, coding for calcium channel Cav3.3, was upregulated in TG after foramen rotundum inflammatory constriction of the trigeminal infraorbital nerve (FRICT-ION) in mice.

#### Tachykinin family

Genes coding for proteins in the tachykinin family have recently been examined in two separate studies.^57,58^ Neurokinin 3 receptor and hemokinin-1 receptor, coded by Tacr3 and Tac4 respectively, are suggested to contribute to development of trigeminal neuropathic pain. Downregulation of Tacr3 worked protective against pain, while upregulation of Tac4 was seen after CFA-injection in mice. The studies suggest that neurokinin 3 receptor and hemokinin-1 receptor are involved in development of trigeminal pain.

Several other genes were proposed in the experimental animal studies, all presented in Table 3.

## Discussion

This systematic review discloses a growing literature on genetic variation in patients with TN, but there are few studies on the prevalence of familial TN. The growing body of genes identified in experimental studies on trigeminal pain may perhaps further underpin a genetic role in TN, even though experimental trigeminal pain and human TN are different conditions.

### Familial trigeminal neuralgia

Several case reports describe families with clustering of TN, but in most studies, there are too few patients to estimate a prevalence of familial TN (Table 1). However, some studies suggest that 1–2% of all TN cases may be a familial form.^13,15–17^ Some of the studies are of older date and investigate a small study population, which may not necessarily make the estimated prevalence transferable to a larger population. Possibly, spare knowledge of genetic involvement in TN may be a challenge, contributing to underreporting of familial TN cases.

### Human studies on genetics in TN

At present, few human studies have addressed a genetic involvement in TN. The most important candidate genes from the human studies may be categorized according to tentative mechanism, namely genes coding for sodium channels, calcium channels and serotonin transporters.

Sodium channels are known therapeutic targets in the treatment of TN, and first line treatment of TN is sodium channel blockers such as carbamazepine or oxcarbazepine.^59^ The responses of nociceptors to stimuli are encoded by action potentials, which are dependent on voltage-gated sodium channels.^60^ Differential expression of sodium channels was observed in one human study included in this systematic review.^14^ The authors suggested that Na_v_1.7 single mutations can cause congenital insensitivity to pain or chronic neuropathic pain syndromes, while co-expression with Na_v_1.8 sustains the initial action potential. Na_v_1.3 is expressed in neurons after injury, and was also upregulated in the patients in this study.

Several human studies^34,35,38^ present sodium channel mutations in patients with TN, suggesting the mutations to increase the excitability of trigeminal ganglion (TG), thus reducing the action potential threshold. These findings make it likely that sodium channels are important for disease pathogenesis in TN, and that mutations in these genes may as well be involved in familial forms of TN. Moreover, the association between sodium channel expression and TN supports the use of sodium channel blockers as treatment of the disease, and provides potential targets for new therapeutic drugs.

Calcium channels are potential therapeutic targets for TN treatment, and calcium channel blockers such as gabapentin are used as alternatives to sodium channel blocker carbamazepine.^5^ Gambeta et al.^36^ investigated a Proline 2455 Histidine mutation in CACNA1A gene, coding for calcium channel Ca_V_2.1, which reduced the calcium-dependent inactivation of the channel. The authors observed a depolarizing shift in the voltage-dependence of activation and inactivation of the channel, as well as strongly reduced calcium-dependent activation of the channel that is consistent with an overall gain of function. They suggested that the depolarizing shift in activation may be more important in neurons with lower firing rates, while the gain of function may have a more profound impact in neurons with high frequency firing. The authors conclude that the changes in Ca_V_2.1 therefore may contribute to the development of TN.

Di Stefano et al.^35^ investigated different mutations in ion channels in TN patients, where whole-exome sequencing analysis identified 41 mutations in ion channels, 7 of them being rare variants of calcium channels. These findings suggest an importance of calcium channels in pathophysiology of TN, thus also possible therapeutic targets in treatment of TN. The authors emphasize that the findings support the need for further study of possible genetic contributions to disease pathogenesis in patients with familial TN.

Serotonin is an important neuromodulator associated with a wide range of physiological effects in the central nervous system, and has been implicated in pain conditions mediated by the trigeminal system.^41^ SLC6A4, a gene coding for serotonin transporter (5-HTT) was investigated in by Cui et al.^41^ The serotonin transporter gene-linked polymorphic region (5-HTTLPR) plays an important role in regulating the concentration of serotonin in brain synapses. The genotype distribution of 5-HTTLPR between TN patients and controls were significantly different, where the TN patients had a higher prevalence of the short-short genotype. The authors concluded that the gene polymorphism was associated with the susceptibility to TN.

Taken together, even though the role of the mentioned genes in familial TN is not yet known, the findings may be an indicator that there is a significant genetic involvement in TN development and/or susceptibility.

### Animal studies on genetic involvement in experimental trigeminal pain

The present systematic review disclosed a steadily increasing number of animal studies examining genetic mechanisms in experimental trigeminal pain. A wide range of genes has been proposed. Some of the genes investigated in the experimental animal studies coincide with the ones investigated in human studies, which strengthens the findings in the human studies. The candidate genes from the animal studies may be categorized into genes coding for sodium channels, potassium channels, TRP channels, purinoceptors, and tachykinin proteins.

Voltage-gated sodium channel genes SCN3A (Na_V_1.3), SCN8A (Na_V_1.6), SCN9A (Na_V_1.7), SCN10A (Na_V_1.8) and SCN11A (Na_V_1.9) were investigated by Liu et al.^61^ and Luiz et al.,^46^ and differential expression of sodium channels implicate that they may be important contributors for development of experimental trigeminal pain. These findings correlate with the conclusion in several human studies^14,33–35,38^ examining the role of sodium channels in TN patients. As previously mentioned, sodium channel blockers carbamazepine and oxcarbazepine are the first line pharmacological treatment in TN, but undesired effects related to the drugs cause withdrawal from treatment in an important percentage of patients.^62^ Vixotrigine, a new sodium channel blocker targeting Na_V_1.7, was recently discovered and has shown efficacy in TN patients.^63^ New drugs with improved analgesic effect and milder adverse effects are desired, and sodium channels identified in this systematic review may be potential targets for new analgesics.

Potassium channels are also suggested to be potential targets for analgesics.^64^ Silencing of Kir4.1, coded by Kcnj10, led to pain-like behavior in one study.^51^ Kcnk18 (coding for TRESK) was investigated in two separate studies^53,54^ where loss of TRESK resulted in mechanical allodynia. Calcium-activated potassium channel BKCa is in another study^55^ proposed to be a potential therapeutic target for trigeminal neuropathic pain. Other potassium channel genes that are proposed to be involved in experimental neuropathic pain are Kcnip3, Kcnj6, Kcnq2 and Kcnq3.^45^ Besides differential expression of these genes in rats after CCI-ION, the specific role of these potassium channel genes in experimental trigeminal neuropathic pain is not further discussed by the authors.

TRP channels have been shown to be expressed in primary afferent nociceptors, where they act as transducers for thermal, chemical and mechanical stimuli.^65^ Demartini et al.^49^ observed an increase of Trpa1 mRNA in the ipsilateral TG and cervical spinal cord after CCI-ION in rats. TRPA1 antagonist ADM_12 significantly reduced the mechanical allodynia, as well as the antagonist abolished the increased levels of TRPA1 and TRPV after CCI-ION in rats. These findings support the hypothesis that TRPA1 and TRPV channels are involved in experimental trigeminal pain. Another study^48^ found that Trpa1 positive mice had a lower pain threshold after CCI-ION, while genetic deletion of Trpa1 reduced pain in CCI-ION mice. Chen et al.^47^ found that Trpa1 knock-out mice did not develop pain, and that inhibitors of the Trpa1 receptor attenuated pain. These findings make it likely that the Trpa1 gene is involved in development of experimental trigeminal neuropathic pain.

Trpv genes, coding for different TRPV channels, were investigated in several studies. Rozas et al.^66^ suggest that overexpression of TNF-α in mice may regulate Cdk5 (cyclin-dependent kinase 5 – a protein involved in pain signaling) activity in TG, inducing sensitization of the TRPV1 channel (ion channel involved in thermal nociception and inflammatory pain). TRPV4 was also considered important for pain transmission in the same study by Chen et al.^47^ where Trpa1 was investigated, and knock-out mice for both genes attenuated pain. As well as an increase in Trpa1, Demartini et al.^49^ also observed an increase in Trpv1 mRNA in the ipsilateral TG and cervical spinal cord in CCI-ION rats.

Further studies on TRP channels in TN patients are needed to determine whether drugs targeting the channels may be effective against TN.

Purinoceptor activation mediates microglia-neuron signaling leading to pain hypersensitivity, and the receptors are promising pharmacological targets in the management of neuropathic pain.^67^ P2x4r gene, coding for a ligand-gated ion channel named P2X purinoceptor 4, was in one study^50^ upregulated after manipulation of the CNV, and blockage of the receptor resulted in an anti-nociceptive effect. P2Y_14_ receptor, another purinoceptor subtype, was upregulated after CFA-injection in mice in another study,^68^ and a P2Y1_14_ receptor antagonist attenuated pain.

Tachykinin proteins are widely distributed within the peripheral and central nervous system and play a well-recognized role as excitatory neurotransmitters.^69^ Cui et al.^57^ observed downregulation of Tacr3, coding for Neurokinin 3 receptor (NK3R), after partial transection of the infraorbital nerve nerve (pT-ION) in mice. The authors suggest that the downregulation plays a protective role in treating allodynia, by suppressing the hyperexcitability in neurons. Another study^58^ observed upregulation of Tac4 gene, encoding Hemokinin-1 (HK-1), concluding that HK-1 participates in pain transmission in the trigeminal system by interacting between sensory neurons and satellite glial cells.

Given the types of manipulation of the trigeminal nerve applied in the experimental studies, the experimental trigeminal pain may be more related to neuropathic trigeminal pain. The CCI-ION is the most frequently used method to establish trigeminal pain in animal studies model, and is not directly transferable to idiopathic cases of TN in humans. Therefore, caution should be made when extrapolating experimental results to human TN. On the other hand, the experimental studies provide important insight, and suggest a stronger genetic involvement in trigeminal pain than previously thought. One mechanism that is repeated in several studies is differential expression of ion channels, or mutations in the genes coding for ion channels. Whether the changes in expression of ion channels indicate genetic involvement in the pathogenesis of trigeminal pain or is primarily involved in pain transmission mechanisms is still not fully determined. Further human studies are warranted to examine whether the genes involved in experimental pain play a role in pathophysiology of human TN.

Several systematic reviews on trigeminal neuralgia and genetics were recently published,^6^,^70--72^ and it is chosen to discuss one of the reviews here, although the article is not included in our results. Smith et al.^71^ performed a systematic review on molecular mechanisms of trigeminal neuralgia. 14 studies were included, with a main focus on ion channels, DREAM (downstream regulatory element antagonist modulator) protein, serotonin transporter, neuregulin-1, and gap junctions. Their conclusion was that researchers have identified multiple genetic and molecular mechanisms that are associated with TN, but that there does not seem to be one specific frontrunner gene, suggesting that predisposition to TN may involve multiple genes. These findings correlate with the results of the current systematic review, where multiple genes have been proposed as possible contributors to TN and to experimental neuropathic trigeminal pain, indicating that the potential genetic mechanisms are complex and involve several genes.

With regard to familial TN, we would comment a study by Hemminki et al.,^73^ describing a pool of 654 patients. Investigation was done on familial risks for siblings who were hospitalized for nerve, nerve root and plexus disorders. Trigeminal neuralgia was suggested to have a familial risk, but there was also seen a correlation between spouses, implying environmental factors to be involved. The study was excluded from this systematic review as the condition of the study population was based on criteria from ICD-10, thereby including other trigeminal nerve conditions than trigeminal neuralgia in particular. As mentioned in the Methods section, other human trigeminal nerve conditions than TN were not to be included in this systematic review.

## Conclusion

From this systematic review, we conclude that genetic factors seem to play a more important role in the pathophysiology of TN than previously assumed. The existing literature on the occurrence of familial TN is sparse and indicates a prevalence of familial form in 1–2% of individuals with TN, but underreporting cannot be excluded. Even though there are rather few human studies on the role of genetic factors in TN, several candidate genes have been proposed. On the other hand, there is an increasing body of studies on experimental trigeminal pain in animals that have identified a number of candidate genes, some of which also have been identified in human studies on TN. We advocate that great caution should be taken when translating experimental findings, probably most relevant for neuropathic trigeminal pain, to mechanisms behind TN in humans. Therefore, the genes identified in animal studies need to be studied in humans as they may be important for TN in humans. In particular, the experimental animal studies highlight the role of genes coding for different ion channels, purinoceptors and tachykinin proteins. From the existing knowledge, the genetic background of TN seems to be complex, involving multiple genes. Nevertheless, further studies on genes involved in TN pathogenesis seems highly warranted.

## Supplemental Material

sj-pdf-1-mpx-10.1177_17448069211016139 - Supplemental material for Trigeminal neuralgia and genetics: A systematic review Click here for additional data file. Supplemental material, sj-pdf-1-mpx-10.1177_17448069211016139 for Trigeminal neuralgia and genetics: A systematic review by Mari Aaroe Mannerak, Aslan Lashkarivand, Per Kristian Eide in Molecular Pain
