# Supplementary material for: Trigeminal neuralgia and genetics: A systematic review
Source: Mol Pain. 2021 May 17;17:17448069211016139. doi: 10.1177/17448069211016139 (PMC8135221; doi:10.1177/17448069211016139)
Supplement: sj-pdf-1-mpx-10.1177_17448069211016139 - Supplemental material for Trigeminal neuralgia and genetics: A systematic review [file sj-pdf-1-mpx-10.1177_17448069211016139.pdf]

Supplementary Material

Trigeminal neuralgia and genetics: A systematic review

Mari Aaroe Mannerak[1], Aslan Lashkarivand[2], Per Kristian Eide[2,3]

[1]Faculty of Medicine, University of Oslo, Oslo, Norway
[2]Department of Neurosurgery, Oslo University Hospital – Rikshospitalet, Oslo, Norway
[3]Institute of Clinical Medicine, Faculty of Medicine, University of Oslo, Oslo, Norway

Corresponding author:
Professor Per Kristian Eide, MD PhD
Dept. of Neurosurgery,
Oslo University Hospital – Rikshospitalet
PB 4950 Nydalen, 0424 OSLO, Norway
Phone: +47 91649419
Fax: +47-23074310
E-mail: p.k.eide@medisin.uio.no

Contents

eAppendix 1. Literature search documentation 3
eAppendix 2. PRISMA 2009 Checklist 7
eAppendix 3. Oxford Centre of Evidence Based Medicine (OCEBM)........................ 9
References 10

eAppendix 1. Literature search documentation

Search strategy documentation for following research question: What is the prevalence of familial TN and which genes are involved in the development of trigeminal neuralgia?

The following databases were searched:

Database	Number of references
Medline (Ovid):	429
Embase (Ovid):	546
Cochrane Library: (Cochrane reviews, CENTRAL)	39
Science citation Index (Web of Science)	318
Total references before duplicate check	1332
Total references after duplicate check	774

All searches are done by Hilde Iren Flaatten, Special librarian at the Medical Library, Rikshospitalet – University of Oslo.

Time spent: 28 hours

Database: Ovid MEDLINE(R) ALL <1946 to January 08, 2021>
Search date: 11.01.2021
Number of references: 429
Search strategy:

1 Trigeminal Neuralgia/ or (Trigeminal Nerve/ and Facial Pain/)
2 ((trigeminal or trigeminus or fifth cranial nerve* or fifth nerve*) adj4 (neuralgia or neuropath* or pain* or ach*)).tw,kf.
3 (prosopalgia* or prosoponeuralgia* or trifacial neuralgia or Tic Douloureux or Fothergill).tw,kf.
4 or/1-3
5 (familial or (famil* adj3 histor*) or (famil* adj3 cluster*) or (famil* adj3 anamnes*) or gene or genes or genetic* or cytogenetic* or genom* or epigenom* or heredit* or heritab* or inherit* or heritage* or autosomal or allel* or nonallel* or nullallel* or haplotype* or haplo type* or chromosom* or chromo som* or exome* or mitochondrial or mito chondrial or heterozygo* or hetero zygo* or hemizygo* or hemi zygo* or zygote or zygos* or homozygo* or homo zygo* or dizygo* or di zygo* or monozygo* or mono zygo* or twin or twins or epistasis or mutat* or mutant* or mutagen* or muta gen* or hypermuta* or hyper muta* or dna* or rna* or microrna* or epigenetic* or genotyp* or geno typ* or phenotyp* or pheno typ* or penetrance or antisense).tw,kf.
6 (SLC6A4 or SCN8A or P2X4R or SCN11A or Cav#2#1 or Cav#2#2 or TRPV1 or GluR2 or GluR3 or Nav19 or Nav 19 or Nav17 or Nav 17 or Nav18 or Nav 18 or Nav13 or Nav 13).tw,kf.
7 exp genetics/ or exp genetic phenomena/ or exp genetic predisposition to disease/ or exp genetic testing/ or exp phenotype/ or exp genotype/ or exp genetic techniques/ or exp chromosome aberrations/ or exp mutagens/ or exp genomic instability/ or genetic markers/ or exp twins/ or exp "antisense elements (genetics)"/
8 5 or 6 or 7
9 4 and 8
10 4 and genetics.fs.
11 9 or 10
12 limit 11 to english language

Database: Embase Classic+Embase <1947 to 2021 January 08>
Search date: 11.01.2021
Number of references: 546
Search strategy:

1 trigeminus neuralgia/ or (trigeminal nerve/ and facial pain/)
2 ((trigeminal or trigeminus or fifth cranial nerve* or fifth nerve*) adj4 (neuralgia or neuropath* or pain* or ach*)).tw,kw.
3 (prosopalgia* or prosoponeuralgia* or trifacial neuralgia or Tic Douloureux or Fothergill).tw,kw.
4 or/1-3
5 (familial or (famil* adj3 histor*) or (famil* adj3 cluster*) or (famil* adj3 anamnes*) or gene or genes or genetic* or cytogenetic* or genom* or epigenom* or heredit* or heritab* or inherit* or heritage* or autosomal or allel* or nonallel* or nullallel* or haplotype* or haplo type* or chromosom* or chromo som* or exome* or mitochondrial or mito chondrial or heterozygo* or hetero zygo* or hemizygo* or hemi zygo* or zygote or zygos* or homozygo* or homo zygo* or dizygo* or di zygo* or monozygo* or mono zygo* or twin or twins or epistasis or mutat* or mutant* or mutagen* or muta gen* or hypermuta* or hyper muta* or dna* or rna* or microrna* or epigenetic* or genotyp* or geno typ* or phenotyp* or pheno typ* or penetrance or antisense).tw,kw.
6 (SLC6A4 or SCN8A or P2X4R or SCN11A or Cav#2#1 or Cav#2#2 or TRPV1 or GluR2 or GluR3 or Nav19 or Nav 19 or Nav17 or Nav 17 or Nav18 or Nav 18 or Nav13 or Nav 13).tw,kw.
7 exp twins/ or genetic association/ or genome-wide association study/ or genetic association study/ or genetic screening/ or exp zygosity/ or genetic parameters/ or exp chromosomal parameters/ or exp "gene and nucleic acid parameters"/ or exp genetic predisposition/ or genetic risk/ or mutation rate/ or penetrance/ or exp inheritance/ or heredity/ or allelism/ or allele/ or genetic association/ or genetic background/ or genetic profile/ or allelic exclusion/ or genetic complementation/ or genetic damage/ or chromosome damage/ or dna damage/ or exp genomic instability/ or exp genetic heterogeneity/ or genetic line/ or substitution line/ or translocation line/ or genetic organization/ or genetic resource/ or genetic stability/ or genetic trait/ or genome/ or genome imprinting/ or genome size/ or genomic fragment/ or human genome/ or mitochondrial genome/ or heritability/ or microsatellite instability/ or mutation/ or chromosome mutation/ or gene mutation/ or allelic imbalance/ or deletion mutant/ or frameshift mutation/ or "gain of function mutation"/ or gene deletion/ or gene disruption/ or gene insertion/ or gene loss/ or indel mutation/ or "loss of function mutation"/ or missense mutation/ or mutator gene/ or nonsense mutation/ or null allele/ or point mutation/ or silent mutation/ or splicing defect/ or genomic instability/ or induced mutation/ or exp mutagenesis/ or mutant/ or mutation rate/ or somatic mutation/ or spontaneous mutation/ or gene frequency/ or gene segregation/ or genetic distance/ or genetic linkage/ or sex linkage/ or x chromosome linkage/ or y chromosome linkage/ or genetic load/ or mutational load/ or genetic marker/ or dna marker/ or marker chromosome/ or marker gene/ or microsatellite marker/ or random amplified microsatellite/ or random amplified polymorphic dna/ or sequence characterized amplified region/ or genetic polymorphism/ or chromosome polymorphism/ or dna polymorphism/ or polymorphic locus/ or protein polymorphism/ or genetic selection/ or exp genotype/ or exp phenotype/ or genotype phenotype correlation/ or exp chromosome aberration/ or exp mutagenic agent/ or exp microRNA/
8 5 or 6 or 7
9 4 and 8
10 limit 9 to conference abstracts
11 9 not 10
12 limit 11 to english language

Database: Science Citation Index (Web of Science)
Search date: 11.01.2021
Number of references: 318
Search strategy:
# 1
TS=((trigeminal OR trigeminus OR "fifth cranial nerve*" OR "fifth nerve*") NEAR/4 (neuralgia OR neuropath* OR pain* OR ach*) OR prosopalgia* OR prosoponeuralgia* OR "trifacial neuralgia" OR "Tic Douloureux" OR Fothergill)
# 2
TS=(familial OR (famil* NEAR/2 histor*) OR (famil* NEAR/2 cluster*) OR (famil* NEAR/2 anamnes*) OR gene OR genes OR genetic* OR cytogenetic* OR genom* OR epigenom* OR heredit* OR heritab* OR inherit* OR heritage* OR autosomal OR allel* OR nonallel* OR nullallel* OR haplotype* OR haplo type* OR chromosom* OR chromo som* OR exome* OR mitochondrial OR mito chondrial OR heterozygo* OR hetero zygo* OR hemizygo* OR hemi zygo* OR zygote OR zygos* OR homozygo* OR homo zygo* OR dizygo* OR di zygo* OR monozygo* OR mono zygo* OR twin OR twins OR epistasis OR mutat* OR mutant* OR mutagen* OR muta gen* OR hypermuta* OR hyper muta* OR dna* OR rna* OR micrORna* OR epigenetic* OR genotyp* OR geno typ* OR phenotyp* OR pheno typ* OR penetrance OR antisense)
(#1 AND #2) AND LANGUAGE: (English) AND DOCUMENT TYPES: (Article)
Indexes=SCI-EXPANDED Timespan=1900-2020

Database: Cochrane Library (Cochrane reviews, CENTRAL)
Search date: 11.01.2021
Number of references: 39
Search strategy:

#1 MeSH descriptor: [Trigeminal Neuralgia] this term only
#2 MeSH descriptor: [Trigeminal Nerve] this term only
#3 MeSH descriptor: [Facial Pain] this term only
#4 #2 AND #3
#5 ((trigeminal or trigeminus or "fifth cranial nerve*" or "fifth nerve*") NEAR/3 (neuralgia or neuropath* or pain* or ach*)):ti,ab,kw
#6 (prosopalgia* or prosoponeuralgia* or trifacial neuralgia or Tic Douloureux or Fothergill):ti,ab,kw
#7 #1 OR #4 OR #5 OR #6
#8 (familial or (family NEAR/2histor*) or (family NEAR/2 cluster*) or (family NEAR/2 anamnes*) or *gene or *genes or *genetic* or cytogenetic* or genom* or epigenom* or heredit* or heritab* or inherit* or heritage* or autosomal or allel* or nonallel* or nullallel* or haplotype* or "haplo type*" or chromosom* or "chromo som*" or exome* or mitochondrial or "mito chondrial" or heterozygo* or "hetero zygo*" or hemizygo* or "hemi zygo*" or zygote or zygos* or homozygo* or "homo zygo*" or dizygo* or "di zygo*" or monozygo* or "mono zygo*" or twin or twins or epistasis or mutat* or mutant* or mutagen* or "muta gen*" or hypermuta* or "hyper muta*" or dna* or rna* or microrna* or epigenetic* or genotyp* or "geno typ*" or phenotyp* or "pheno typ*" or penetrance or antisense):ti,ab,kw (Word variations have been searched)
#9 (SLC6A4 or SCN8A or P2X4R or SCN11A or Cav?2?1 or Cav?2?2 or TRPV1 or GluR2 or GluR3 or Nav19 or "Nav 19" or Nav17 or "Nav 17" or Nav18 or "Nav 18" or "Nav 13" or Nav13):ti,ab,kw
#10 MeSH descriptor: [Genetics] explode all trees
#11 MeSH descriptor: [Genetic Phenomena] explode all trees
#12 MeSH descriptor: [Genetic Predisposition to Disease] explode all trees
#13 MeSH descriptor: [Genetic Techniques] explode all trees

#14 MeSH descriptor: [Genetic Testing] explode all trees
#15 MeSH descriptor: [Phenotype] explode all trees
#16 MeSH descriptor: [Genotype] explode all trees
#17 MeSH descriptor: [Chromosome Aberrations] explode all trees
#18 MeSH descriptor: [Mutagens] explode all trees
#19 MeSH descriptor: [Genomic Instability] explode all trees
#20 MeSH descriptor: [Genetic Markers] this term only
#21 MeSH descriptor: [Twins] explode all trees
#22 MeSH descriptor: [Antisense Elements (Genetics)] explode all trees
#23 {OR #8-#22}
#24 #7 AND #23

eAppendix 2. PRISMA 2009 Checklist

PRISMA 2009 Checklist: Checklist of items to include when reporting a systematic review

Section/topic	#	Checklist item	Reported on page #
Title	1	Identify the report as a systematic review, meta-analysis, or both.	1
**ABSTRACT**			
Structured summary	2	Provide a structured summary including, as applicable: background; objectives; data sources; study eligibility criteria, participants, and interventions; study appraisal and synthesis methods; results; limitations; conclusions and implications of key findings; systematic review registration number.	3
**INTRODUCTION**			
Rationale	3	Describe the rationale for the review in the context of what is already known.	4
Objectives	4	Provide an explicit statement of questions being addressed with reference to participants, interventions, comparisons, outcomes, and study design (PICOS).	4
**METHODS**			
Protocol and registration	5	Indicate if a review protocol exists, if and where it can be accessed (e.g., Web address), and, if available, provide registration information including registration number.	5
Eligibility criteria	6	Specify study characteristics (e.g., PICOS, length of follow-up) and report characteristics (e.g., years considered, language, publication status) used as criteria for eligibility, giving rationale.	5
Information sources	7	Describe all information sources (e.g., databases with dates of coverage, contact with study authors to identify additional studies) in the search and date last searched.	5
Search	8	Present full electronic search strategy for at least one database, including any limits used, such that it could be repeated.	Suppl. material
Study selection	9	State the process for selecting studies (i.e., screening, eligibility, included in systematic review, and, if applicable, included in the meta-analysis).	5
Data collection process	10	Describe method of data extraction from reports (e.g., piloted forms, independently, in duplicate) and any processes for obtaining and confirming data from investigators.	6
Data items	11	List and define all variables for which data were sought (e.g., PICOS, funding sources) and any assumptions and simplifications made.	6-7
Risk of bias in individual studies	12	Describe methods used for assessing risk of bias of individual studies (including specification of whether this was done at the study or outcome level), and how this information is to be used in any data synthesis.	6 + Suppl. material
Summary measures	13	State the principal summary measures (e.g., risk ratio, difference in means).	N/A
Synthesis of results	14	Describe the methods of handling data and combining results of studies, if done, including measures of consistency (e.g., I^2) for each meta-analysis.	8-13

Risk of bias across studies	15	Specify any assessment of risk of bias that may affect the cumulative evidence (e.g., publication bias, selective reporting within studies).	N/A
Additional analyses	16	Describe methods of additional analyses (e.g., sensitivity or subgroup analyses, meta-regression), if done, indicating which were pre-specified.	N/A
**RESULTS**			
Study selection	17	Give numbers of studies screened, assessed for eligibility, and included in the review, with reasons for exclusions at each stage, ideally with a flow diagram.	8 and Fig. 1
Study characteristics	18	For each study, present characteristics for which data were extracted (e.g., study size, PICOS, follow-up period) and provide the citations.	Table 1-3
Risk of bias within studies	19	Present data on risk of bias of each study and, if available, any outcome level assessment (see item 12).	6 + Suppl. material
Results of individual studies	20	For all outcomes considered (benefits or harms), present, for each study: (a) simple summary data for each intervention group (b) effect estimates and confidence intervals, ideally with a forest plot.	N/A
Synthesis of results	21	Present results of each meta-analysis done, including confidence intervals and measures of consistency.	N/A
Risk of bias across studies	22	Present results of any assessment of risk of bias across studies (see Item 15).	N/A
Additional analysis	23	Give results of additional analyses, if done (e.g., sensitivity or subgroup analyses, meta-regression [see Item 16]).	N/A
**DISCUSSION**			
Summary of evidence	24	Summarize the main findings including the strength of evidence for each main outcome; consider their relevance to key groups (e.g., healthcare providers, users, and policy makers).	13-19
Limitations	25	Discuss limitations at study and outcome level (e.g., risk of bias), and at review-level (e.g., incomplete retrieval of identified research, reporting bias).	19-20
Conclusions	26	Provide a general interpretation of the results in the context of other evidence, and implications for future research.	19-20
**FUNDING**			
Funding	27	Describe sources of funding for the systematic review and other support (e.g., supply of data); role of funders for the systematic review.	20

N/A = not applicable
Not all items are applicable as the current systematic review was not aimed to be an intervention study.

From: Moher D, Liberati A, Tetzlaff J, Altman DG, The PRISMA Group (2009). Preferred Reporting Items for Systematic Reviews and Meta-Analyses: The PRISMA Statement. PLoS Med 6(7): e1000097. doi:10.1371/journal.pmed1000097

eAppendix 3. Oxford Centre of Evidence Based Medicine (OCEBM)

Levels of Evidence

Manuscript sections	Studies included by evidence level
Familial trigeminal neuralgia	- 23 Level 5 studies [1-33]
Human studies on trigeminal neuralgia and genetics	- 4 level 4 studies [24, 25, 30, 31]<br>- 6 level 5 studies [23, 26-29, 32]
Experimental animal studies on trigeminal neuralgia and genetics	- 38 Level 5 studies [34-71]

Explanation of levels:
1. Systematic review with meta-analysis of RCTs
2. RCT or observational study of high quality
3. Non-randomized follow-up/observational study
4. Case series or case-control studies
5. Mechanism-based reasoning (expert opinion, case report, animal or laboratory studies)

OCEBM Levels of Evidence Working Group*. "The Oxford 2011 Levels of Evidence". Oxford Centre for Evidence-Based Medicine. http://www.cebm.net/index.aspx?o=5653

** OCEBM Table of Evidence Working Group = Jeremy Howick, Iain Chalmers (James Lind Library), Paul Glasziou, Trish Greenhalgh, Carl Heneghan, Alessandro Liberati, Ivan Moschetti, Bob Phillips, Hazel Thornton, Olive Goddard and Mary Hodgkinson*

References

1. Allan W. Familial occurrence of tic douloureux. *Arch Neurol Psychiatry* 1938; 40: 1019-1020. Article. DOI: 10.1001/archneurpsyc.1938.02270110173014.
2. Auld AW and Buermann A. Trigeminal Neuralgia in Six Members of One Generation. *Arch Neurol* 1965; 13: 194.
3. Braga FM, Bonatelli Ade P, Suriano I and Canteras M. Familial trigeminal neuralgia. *Surg Neurol* 1986; 26: 405-408. Case Reports.
4. Cervera-Martinez C, Martinez-Manrique JJ and Revuelta-Gutierrez R. Surgical Management of Familial Trigeminal Neuralgia With Different Inheritance Patterns: A Case Report. *Front Neurol* 2018; 9. Article. DOI: 10.3389/fneur.2018.00316.
5. Coffey RJ and Fromm GH. Familial trigeminal neuralgia and Charcot-Marie-Tooth neuropathy. Report of two families and review. *Surg Neurol* 1991; 35: 49-53. Case Reports Review.
6. Cruse RP, Conomy JP, Wilbourn AJ and Hanson MR. HEREDITARY HYPERTROPHIC NEUROPATHY COMBINING FEATURES OF TIC DOULOUREUX, CHARCOT-MARIE-TOOTH DISEASE, AND DEAFNESS. *Cleve Clin Q* 1977; 44: 107-111. Article. DOI: 10.3949/ccjm.44.3.107.
7. Daly RF and Sajor EE. INHERITED TIC DOULOUREUX. *Neurology* 1973; 23: 937-939. Article. DOI: 10.1212/wnl.23.9.937.
8. Denu RA, Rosenberg SA and Howard SP. Familial Trigeminal Neuralgia Treated with Stereotactic Radiosurgery: A Case Report and Literature Review. *J* 2017; 6: 149-152. DOI: https://dx.doi.org/10.1007/s13566-017-0300-0.
9. DiCorato MP and Pierce BA. Familial trigeminal neuralgia. *South Med J* 1985; 78: 353-354. Case Reports.
10. Duff JM, Spinner RJ, Lindor NM, Dodick DW and Atkinson JL. Familial trigeminal neuralgia and contralateral hemifacial spasm. *Neurology* 1999; 53: 216-218. Case Reports.
11. Ebner FH, Tatagiba M and Roser F. Familial trigeminal neuralgia--microsurgical experience and psychological observations. *Acta Neurochir (Wien)* 2010; 152: 381-382. Case Reports
Letter. DOI: https://dx.doi.org/10.1007/s00701-009-0413-3.
12. Fernandez Rodriguez B, Simonet C, Cerdan DM, Morollon N, Guerrero P, Tabernero C and Duarte J. Familial classic trigeminal neuralgia. *Neurologia* 2019; 34: 229-233. DOI: https://dx.doi.org/10.1016/j.nrl.2016.12.004.
13. Fleetwood IG, Innes AM, Hansen SR and Steinberg GK. Familial trigeminal neuralgia - Case report and review of the literature. *J Neurosurg* 2001; 95: 513-517. Article. DOI: 10.3171/jns.2001.95.3.0513.
14. Gupta V, Singh AK, Kumar S and Sinha S. Familial trigeminal neuralgia. *Neurol India* 2002; 50: 87-89. Article.
15. Herzberg L. FAMILIAL TRIGEMINAL NEURALGIA. *Arch Neurol* 1980; 37: 285-286. Article. DOI: 10.1001/archneur.1980.00500540063007.
16. Kirkpatrick DB. Familial trigeminal neuralgia: case report. *Neurosurgery* 1989; 24: 758-761. Case Reports.
17. Knuckey NW and Gubbay SS. Familial trigeminal and glossopharyngeal neuralgia. *Clin Exp Neurol* 1979; 16: 315-319. Case Reports
Research Support, Non-U.S. Gov't.
18. Mereaux JL, Lefaucheur R, Hebant B, Guegan-Massardier E and Grangeon L. Trigeminal Neuralgia and Charcot-Marie-Tooth Disease: An Intriguing Association. Lessons

From a Large Family Case Report and Review of Literature. *Headache* 2019; 59: 1074-1079. DOI: https://dx.doi.org/10.1111/head.13576.
19. Savica R, Lagana A, Siracusano R, Calabro RS, Ferlazzo E and Musolino R. Idiopathic familial trigeminal neuralgia: a case report. *Neurol Sci* 2007; 28: 196-198. Article. DOI: 10.1007/s10072-007-0820-2.
20. Smyth P, Greenough G and Stommel E. Familial trigeminal neuralgia: case reports and review of the literature. *Headache* 2003; 43: 910-915. Case Reports
Review.
21. Testa D, Milanese C, La Mantia L, Mastrangelo M, Crenna P and Negri S. Familial trigeminal neuralgia in Charcot-Marie-Tooth disease. *J Neurol* 1981; 225: 283-287. Case Reports.
22. Zhang L, Liang JF and Yu YB. Familial Idiopathic Cranial Neuropathy in a Chinese Family. *Turk Neurosurg* 2016; 26: 449-451. Article. DOI: 10.5137/1019-5149.Jtn.7876-13.3.
23. Caress JB, Lewis JA, Pinyan CW and Lawson VH. A charcot-marie-tooth type 1B kindred associated with hemifacial spasm and trigeminal neuralgia. *Muscle Nerve* 2019; 60: 62-66. Article. DOI: 10.1002/mus.26478.
24. Costa GMF, Rocha LPC, Siqueira S, Moreira PR and Almeida-Leite CM. No Association of Polymorphisms in Nav1.7 or Nerve Growth Factor Receptor Genes with Trigeminal Neuralgia. *Pain Med* 2019; 20: 1362-1369. DOI: https://dx.doi.org/10.1093/pm/pny191.
25. Cui W, Yu X and Zhang H. The serotonin transporter gene polymorphism is associated with the susceptibility and the pain severity in idiopathic trigeminal neuralgia patients. *J Headache Pain* 2014; 15: 42. Research Support, Non-U.S. Gov't. DOI: https://dx.doi.org/10.1186/1129-2377-15-42.
26. Di Lorenzo C, Daverio A, Pasqualetti P, Coppola G, Giannoudas I, Barone Y, Grieco GS, Niolu C, Pascale E, Santorelli FM, Nicoletti F, Pierelli F, Siracusano A, Seri S and Di Lorenzo G. The upstream Variable Number Tandem Repeat polymorphism of the monoamine oxidase type A gene influences trigeminal pain-related evoked responses. *Eur J Neurosci* 2014; 39: 501-507. Article. DOI: 10.1111/ejn.12458.
27. Di Stefano G, Yuan JH, Cruccu G, Waxman SG, Dib-Hajj SD and Truini A. Familial trigeminal neuralgia - a systematic clinical study with a genomic screen of the neuronal electrogenisome. *Cephalalgia* 2020; 40: 767-777. DOI: https://dx.doi.org/10.1177/0333102419897623.
28. Gambeta E, Gandini MA, Souza IA, Ferron L and Zamponi GW. A CACNA1A variant associated with trigeminal neuralgia alters the gating of Cav2.1 channels. *Mol Brain* 2021; 14: 4. DOI: https://dx.doi.org/10.1186/s13041-020-00725-y.
29. Jin Y, Lu HB, Liong E, Lau TY and Tipoe GL. Transcriptional mRNA of BMP-2, 3, 4 and 5 in trigeminal nerve, benign and malignant peripheral nerve sheath tumors. *Histol Histopathol* 2001; 16: 1013-1019. Research Support, Non-U.S. Gov't. DOI: https://dx.doi.org/10.14670/HH-16.1013.
30. Li X, Wang D, Zhou J, Yan Y and Chen L. Evaluation of circulating microRNA expression in patients with trigeminal neuralgia: An observational study. *Medicine* 2020; 99: e22972. Observational Study. DOI: https://dx.doi.org/10.1097/MD.0000000000022972.
31. Siqueira SR, Alves B, Malpartida HM, Teixeira MJ and Siqueira JT. Abnormal expression of voltage-gated sodium channels Nav1.7, Nav1.3 and Nav1.8 in trigeminal neuralgia. *Neuroscience* 2009; 164: 573-577. Journal Article; Randomized Controlled Trial. DOI: 10.1016/j.neuroscience.2009.08.037.

32. Tanaka BS, Zhao P, Dib-Hajj FB, Morisset V, Tate S, Waxman SG and Dib-Hajj SD. A gain-of-function mutation in Nav1.6 in a case of trigeminal neuralgia. *Molecular medicine (Cambridge, Mass)* 2016; 22: 338-348. Journal: Article. DOI: 10.2119/molmed.2016.00131.
33. Harris W. An analysis of 1,433 cases of paroxysmal trigeminal neuralgia (trigeminal-tic) and the end-results of gasserian alcohol injection. *Brain* 1940; 63: 209-224. DOI: 10.1093/brain/63.3.209.
34. Aczel T, Kecskes A, Kun J, Szenthe K, Banati F, Szathmary S, Herczeg R, Urban P, Gyenesei A, Gaszner B, Helyes Z and Bolcskei K. Hemokinin-1 Gene Expression Is Upregulated in Trigeminal Ganglia in an Inflammatory Orofacial Pain Model: Potential Role in Peripheral Sensitization. *Int J Mol Sci* 2020; 21: 22. DOI: https://dx.doi.org/10.3390/ijms21082938.
35. Aczel T, Kun J, Szoke E, Rauch T, Junttila S, Gyenesei A, Bolcskei K and Helyes Z. Transcriptional Alterations in the Trigeminal Ganglia, Nucleus and Peripheral Blood Mononuclear Cells in a Rat Orofacial Pain Model. *Front Mol Neurosci* 2018; 11. Article. DOI: 10.3389/fnmol.2018.00219.
36. Aita M, Byers MR, Chavkin C and Xu M. Trigeminal injury causes kappa opioid-dependent allodynic, glial and immune cell responses in mice. *Mol Pain* 2010; 6. Article. DOI: 10.1186/1744-8069-6-8.
37. Benedet T, Gonzalez P, Oliveros JC, Dopazo JM, Ghimire K, Palczewska M, Mellstrom B and Naranjo JR. Transcriptional repressor DREAM regulates trigeminal noxious perception. *J Neurochem* 2017; 141: 544-552. Article. DOI: 10.1111/jnc.13584.
38. Chen ML, Lin K and Lin SK. NLRP3 inflammasome signaling as an early molecular response is negatively controlled by miR-186 in CFA-induced prosopalgia mice. *Braz J Med Biol Res* 2018; 51. Article. DOI: 10.1590/1414-431x20187602.
39. Chen Y, Kanju P, Fang Q, Lee SH, Parekh PK, Lee W, Moore C, Brenner D, Gereau RWt, Wang F and Liedtke W. TRPV4 is necessary for trigeminal irritant pain and functions as a cellular formalin receptor. *Pain* 2014; 155: 2662-2672. Research Support, N.I.H., Extramural Research Support, Non-U.S. Gov't. DOI: https://dx.doi.org/10.1016/j.pain.2014.09.033.
40. Cui WQ, Chu YX, Xu F, Chen T, Gao L, Feng Y, Hu XM, Yang W, Du LX, Zhang WW, Mao-Ying QL, Mi WL and Wang YQ. Calcium Channel alpha 2 delta 1 Subunit Mediates Secondary Orofacial Hyperalgesia Through PKC-TRPA1/Gap Junction Signaling. *J Pain* 2020; 21: 238-257. Article. DOI: 10.1016/j.jpain.2019.08.012.
41. Cui WQ, Zhang WW, Chen T, Li Q, Xu F, Mao-Ying QL, Mi WL, Wang YQ and Chu YX. Tacr3 in the lateral habenula differentially regulates orofacial allodynia and anxiety-like behaviors in a mouse model of trigeminal neuralgia. *Acta Neuropathol Commun* 2020; 8: 44. Research Support, Non-U.S. Gov't. DOI: https://dx.doi.org/10.1186/s40478-020-00922-9.
42. Daiutolo BV, Tyburski A, Clark SW and Elliott MB. Trigeminal Pain Molecules, Allodynia, and Photosensitivity Are Pharmacologically and Genetically Modulated in a Model of Traumatic Brain Injury. *J Neurotrauma* 2016; 33: 748-760. Article. DOI: 10.1089/neu.2015.4087.
43. Demartini C, Greco R, Zanaboni AM, Francesconi O, Nativi C, Tassorelli C and Deseure K. Antagonism of Transient Receptor Potential Ankyrin Type-1 Channels as a Potential Target for the Treatment of Trigeminal Neuropathic Pain: Study in an Animal Model. *Int J Mol Sci* 2018; 19. Article. DOI: 10.3390/ijms19113320.
44. Dong W, Jin SC, Allocco A, Zeng X, Sheth AH, Panchagnula S, Castonguay A, Lorenzo LE, Islam B, Brindle G, Bachand K, Hu J, Sularz A, Gaillard J, Choi J, Dunbar A, Nelson-Williams C, Kiziltug E, Furey CG, Conine S, Duy PQ, Kundishora AJ, Loring E, Li B, Lu Q, Zhou G, Liu W, Li X, Sierant MC, Mane S, Castaldi C, Lopez-Giraldez F, Knight JR, Sekula RF, Jr., Simard JM,

Eskandar EN, Gottschalk C, Moliterno J, Gunel M, Gerrard JL, Dib-Hajj S, Waxman SG, Barker FG, 2nd, Alper SL, Chahine M, Haider S, De Koninck Y, Lifton RP and Kahle KT. Exome Sequencing Implicates Impaired GABA Signaling and Neuronal Ion Transport in Trigeminal Neuralgia. *iScience* 2020; 23: 101552. DOI: https://dx.doi.org/10.1016/j.isci.2020.101552.

45. Guo Z, Qiu CS, Jiang X, Zhang J, Li F, Liu Q, Dhaka A and Cao YQ. TRESK K⁺ Channel Activity Regulates Trigeminal Nociception and Headache. *eNeuro* 2019; 6. Research Support, Non-U.S. Gov't
Research Support, N.I.H., Extramural. DOI: https://dx.doi.org/10.1523/ENEURO.0236-19.2019.

46. Hanstein R, Hanani M, Scemes E and Spray DC. Glial pannexin1 contributes to tactile hypersensitivity in a mouse model of orofacial pain. *Sci Rep* 2016; 6. Article. DOI: 10.1038/srep38266.

47. Jiang BC, Zhang J, Wu B, Jiang M, Cao H, Wu H and Gao YJ. G protein-coupled receptor GPR151 is involved in trigeminal neuropathic pain via the induction of Gbetagamma/ERK-mediated neuroinflammation in the trigeminal ganglion. *Pain* 2020; 23: 23. DOI: https://dx.doi.org/10.1097/j.pain.0000000000002156.

48. Korczeniewska OA, Husain S, Khan J, Eliav E, Soteropoulos P and Benoliel R. Differential gene expression in trigeminal ganglia of male and female rats following chronic constriction of the infraorbital nerve. *European Journal of Pain* 2018; 22: 875-888. Article. DOI: 10.1002/ejp.1174.

49. Korczeniewska OA, Katzmann Rider G, Gajra S, Narra V, Ramavajla V, Chang YJ, Tao Y, Soteropoulos P, Husain S, Khan J, Eliav E and Benoliel R. Differential gene expression changes in the dorsal root versus trigeminal ganglia following peripheral nerve injury in rats. *European Journal of Pain* 2020; 24: 967-982. DOI: https://dx.doi.org/10.1002/ejp.1546.

50. Lee GW, Son JY, Lee AR, Ju JS, Bae YC and Ahn DK. Central VEGF-A pathway plays a key role in the development of trigeminal neuropathic pain in rats. *Mol Pain* 2019; 15: 1744806919872602. Research Support, Non-U.S. Gov't. DOI: https://dx.doi.org/10.1177/1744806919872602.

51. Li L, Yao L, Wang F and Zhang Z. Knock-down of JAK2 and PTEN on pain behavior in rat model of trigeminal neuropathic pain. *Gene* 2019; 719: 144080. DOI: https://dx.doi.org/10.1016/j.gene.2019.144080.

52. Li Q, Ma TL, Qiu YQ, Cui WQ, Chen T, Zhang WW, Wang J, Mao-Ying QL, Mi WL, Wang YQ and Chu YX. Connexin 36 Mediates Orofacial Pain Hypersensitivity Through GluK2 and TRPA1. *Neurosci Bull* 2020; 36: 1484-1499. DOI: https://dx.doi.org/10.1007/s12264-020-00594-4.

53. Li Y, Jiao H, Ren W and Ren F. TRESK alleviates trigeminal neuralgia induced by infraorbital nerve chronic constriction injury in rats. *Mol Pain* 2019; 15: 1744806919882511. Research Support, Non-U.S. Gov't. DOI: https://dx.doi.org/10.1177/1744806919882511.

54. Lin J, Zhang YY, Liu F, Fang XY, Liu MK, Huang CL, Wang H, Liao DQ, Zhou C and Shen JF. The P2Y₁₄ receptor in the trigeminal ganglion contributes to the maintenance of inflammatory pain. *Neurochem Int* 2019; 131: 104567. Research Support, Non-U.S. Gov't. DOI: https://dx.doi.org/10.1016/j.neuint.2019.104567.

55. Liu C, Zhang Y, Liu Q, Jiang L, Li M, Wang S, Long T, He W, Kong X, Qin G, Chen L, Zhang Y and Zhou J. P2X4-receptor participates in EAAT3 regulation via BDNF-TrkB signaling in a model of trigeminal allodynia. *Mol Pain* 2018; 14: 1744806918795930. Research Support, Non-U.S. Gov't. DOI: https://dx.doi.org/10.1177/1744806918795930.

56. Liu CY, Lu ZY, Li N, Yu LH, Zhao YF and Ma B. The role of large-conductance, calcium-activated potassium channels in a rat model of trigeminal neuropathic pain. *Cephalalgia* 2015; 35: 16-35. Research Support, Non-U.S. Gov't. DOI: https://dx.doi.org/10.1177/0333102414534083.
57. Liu MX, Zhong J, Xia L, Dou NN and Li ST. IL-6 contributes to Na_v1.3 up-regulation in trigeminal nerve following chronic constriction injury. *Neurol Res* 2020; 42: 504-514. DOI: https://dx.doi.org/10.1080/01616412.2020.1747719.
58. Luiz AP, Kopach O, Santana-Varela S and Wood JN. The role of Na<inf>v</inf>1.9 channel in the development of neuropathic orofacial pain associated with trigeminal neuralgia. *Mol Pain* 2015; 11. DOI: http://dx.doi.org/10.1186/s12990-015-0076-4.
59. Luiz AP, Schroeder SD, Rae GA, Calixto JB and Chichorro JG. Contribution and interaction of kinin receptors and dynorphin A in a model of trigeminal neuropathic pain in mice. *Neuroscience* 2015; 300: 189-200. Research Support, Non-U.S. Gov't. DOI: https://dx.doi.org/10.1016/j.neuroscience.2015.05.015.
60. Ma F, Zhang L and Westlund KN. Trigeminal nerve injury ErbB3/ErbB2 promotes mechanical hypersensitivity. *Anesthesiology* 2012; 117: 381-388. Research Support, N.I.H., Extramural
Research Support, Non-U.S. Gov't. DOI: https://dx.doi.org/10.1097/ALN.0b013e3182604b2b.
61. Miyamoto M, Tsuboi Y, Takamiya K, Huganir RL, Kondo M, Shinoda M, Oi Y and Iwata K. Involvement of GluR2 and GluR3 subunit C-termini in the trigeminal spinal subnucleus caudalis and C1-C2 neurons in trigeminal neuropathic pain. *Neurosci Lett* 2011; 491: 8-12. Research Support, N.I.H., Extramural
Research Support, Non-U.S. Gov't. DOI: https://dx.doi.org/10.1016/j.neulet.2010.12.060.
62. Montera M, Goins A, Cmarko L, Weiss N, Westlund KN and Alles SRA. Trigeminal neuropathic pain is alleviated by inhibition of Ca_v3.3 T-type calcium channels in mice. *Channels* 2021; 15: 31-37. DOI: https://dx.doi.org/10.1080/19336950.2020.1859248.
63. Poh KW, Yeo JF, Stohler CS and Ong WY. Comprehensive Gene Expression Profiling in the Prefrontal Cortex Links Immune Activation and Neutrophil Infiltration to Antinociception. *J Neurosci* 2012; 32: 35-45. Article. DOI: 10.1523/jneurosci.2389-11.2012.
64. Rozas P, Lazcano P, Pina R, Cho A, Terse A, Pertusa M, Madrid R, Gonzalez-Billault C, Kulkarni AB and Utreras E. Targeted overexpression of tumor necrosis factor-alpha increases cyclin-dependent kinase 5 activity and TRPV1-dependent Ca2+ influx in trigeminal neurons. *Pain* 2016; 157: 1346-1362. Article. DOI: 10.1097/j.pain.0000000000000527.
65. Trevisan G, Benemei S, Materazzi S, De Logu F, De Siena G, Fusi C, Fortes Rossato M, Coppi E, Marone IM, Ferreira J, Geppetti P and Nassini R. TRPA1 mediates trigeminal neuropathic pain in mice downstream of monocytes/macrophages and oxidative stress. *Brain* 2016; 139: 1361-1377. Research Support, Non-U.S. Gov't. DOI: https://dx.doi.org/10.1093/brain/aww038.
66. Tzabazis AZ, Klukinov M, Feliciano DP, Wilson SP and Yeomans DC. Gene therapy for trigeminal pain in mice. *Gene Ther* 2014; 21: 422-426. Article. DOI: 10.1038/gt.2014.14.
67. Vit JP, Ohara PT, Bhargava A, Kelley K and Jasmin L. Silencing the kir4.1 potassium channel subunit in satellite glial cells of the rat trigeminal ganglion results in pain-like behavior in the absence of nerve injury. *J Neurosci* 2008; 28: 4161-4171. Article. DOI: 10.1523/jneurosci.5053-07.2008.
68. Wang X, Wang H, Zhang T, He M, Liang H, Wang H, Xu L, Chen S and Xu M. Inhibition of MicroRNA-195 Alleviates Neuropathic Pain by Targeting Patched1 and Inhibiting SHH

Signaling Pathway Activation. *Neurochem Res* 2019; 44: 1690-1702. DOI: https://dx.doi.org/10.1007/s11064-019-02797-2.
69. Xu M, Yan Y, Zhu M, Wang Z, Zhang X and Zhang D. Effects of long non-coding RNA Gm14461 on pain transmission in trigeminal neuralgia. *J Inflamm* 2020; 17: 1. DOI: https://dx.doi.org/10.1186/s12950-019-0231-1.
70. Xu W, Zhang J, Wang Y, Wang L and Wang X. Changes in the expression of voltage-gated sodium channels Nav1.3, Nav1.7, Nav1.8, and Nav1.9 in rat trigeminal ganglia following chronic constriction injury. *Neuroreport* 2016; 27: 929-934. Research Support, Non-U.S. Gov't. DOI: https://dx.doi.org/10.1097/WNR.0000000000000632.
71. Zhao LX, Jiang M, Bai XQ, Cao DL, Wu XB, Zhang J, Guo JS, Chen TT, Wang J, Wu H, Gao YJ and Zhang ZJ. TLR8 in the Trigeminal Ganglion Contributes to the Maintenance of Trigeminal Neuropathic Pain in Mice. *Neuroscience Bulletin* 2020. DOI: http://dx.doi.org/10.1007/s12264-020-00621-4.
